# Glycosylation: mechanisms, biological functions and clinical implications

**DOI:** 10.1038/s41392-024-01886-1

**Published:** 2024-08-05

**Authors:** Mengyuan He, Xiangxiang Zhou, Xin Wang

**Affiliations:** 1 Department of Hematology, Shandong Provincial Hospital, Shandong University, Jinan, Shandong 250021 China; 2 Department of Hematology, Shandong Provincial Hospital Affiliated to Shandong First Medical University, Jinan, Shandong 250021 China; 3 National Clinical Research Center for Hematologic Diseases, the First Affiliated Hospital of Soochow University, Suzhou, 251006 China; 4 Taishan Scholars Program of Shandong Province, Jinan, Shandong 250021 China; 5 Branch of National Clinical Research Center for Hematologic Diseases, Jinan, Shandong 250021 China

**Keywords:** Haematological cancer, Epigenetics, Tumour biomarkers, Drug development

## Abstract

Protein post-translational modification (PTM) is a covalent process that occurs in proteins during or after translation through the addition or removal of one or more functional groups, and has a profound effect on protein function. Glycosylation is one of the most common PTMs, in which polysaccharides are transferred to specific amino acid residues in proteins by glycosyltransferases. A growing body of evidence suggests that glycosylation is essential for the unfolding of various functional activities in organisms, such as playing a key role in the regulation of protein function, cell adhesion and immune escape. Aberrant glycosylation is also closely associated with the development of various diseases. Abnormal glycosylation patterns are closely linked to the emergence of various health conditions, including cancer, inflammation, autoimmune disorders, and several other diseases. However, the underlying composition and structure of the glycosylated residues have not been determined. It is imperative to fully understand the internal structure and differential expression of glycosylation, and to incorporate advanced detection technologies to keep the knowledge advancing. Investigations on the clinical applications of glycosylation focused on sensitive and promising biomarkers, development of more effective small molecule targeted drugs and emerging vaccines. These studies provide a new area for novel therapeutic strategies based on glycosylation.

## Introduction

Protein post-translational modification (PTM) is a covalent alteration occurring during or after protein synthesis, often by adding or removing functional groups, profoundly impacting protein function.^1^ More than 300 different PTMs have been identified, including methylation, ubiquitination, acetylation, phosphorylation and glycosylation. Among them, glycosylation is one of the richest and most diverse PTMs, and there are many different types of PTMs in human body, mainly including *N*-glycosylation with asparagine (Asn) linkage, *O*-glycosylation with serine (Ser) and threonine (Thr) linkage, *C*-glycosylation with tryptophan (Trp) linkage, and glycosylphosphatidylinositol (GPI)-anchored attachment, which shows great structural changes.^2^ The glycosylation process begins with the initial transfer of glycosyl in the cell’s endoplasmic reticulum (ER), followed by its entry into the Golgi apparatus, where a range of glycans are added to facilitate glycan maturation, involving numerous glycosyltransferases (GTs) and glycosidases in the process.^3^

The glycosylation pathway produces different protein forms of modified proteins that play a role in many biological functions. Diverse cell adhesion molecules exhibit significant alterations in glycosylation is exhibited in cell adhesion molecules, which are vital for cancer progression, tumor metastasis, and immune evasion.^4^ The significance of glycosylation in signaling pathways, including the complex modulation of signal transduction within the transforming growth factor β (TGF-β) pathway, cannot be understated.^5^ Abnormal glycosylation leads to protein malfunction and disruption of biological processes that can lead to serious diseases. Up-regulation of the glycosyltransferase β-1,3-*N*-acetylglucosaminyl transferase leads to abnormal levels of programmed cell death molecule ligand 1 (PD-L1) glycosylation, which has been implicated in the development of triple-negative breast cancer (TNBC).^6^ Blockade of *N*-glycosylation of sterol regulatory element-binding protein cleavage-activating protein improves epidermal growth factor receptor (EGFR) VIII-driven glioblastoma growth.^7^ The importance of glycosylation in normal physiological functions and diseases in the human body has revealed the need to explore its mechanisms in depth and investigate more effective and tolerable strategies to stop disease production and improve patient prognosis.

**Fig. 1:**
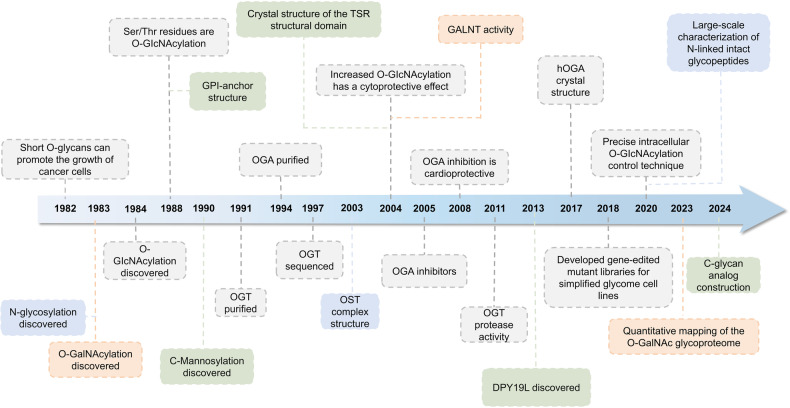
Timeline of the four major glycosylations development and further investigation. In the early 1980s, there was a preliminary understanding of glycosylation. As the research progressed, the corresponding initiating enzymes and chemical structures were gradually deciphered and recognized. For *N*-glycosylation, the determination of the Asn-X-Thr/Ser consensus sequence and the structure of the OST complex subunit and some accessory subunits around 2000 led to the in-depth study. Years later, *N*-linked intact glycopeptides in human serum were identified on a large scale using HILIC enrichment and spectral library searches, and the relationship between *N*-glycosylation levels and glucose metabolic stress, iron apoptosis, and so on were found. *O*-glycosylation is mainly classified into two types: *O*-GlcNAcylation and *O*-GalNAcylation. Since the 2010s, the close connection between *O*-glycosylation and physiological processes such as inflammatory response, immune escape, viral infection, cell adhesion, metastasis, apoptosis, etc. has been continuously discovered. Technological breakthroughs around 2020 have also led to the emergence of quantitative determination of maps of the *O*-glycosylated proteome and the development of gene editing libraries. The understanding of *C*-glycosylation began in the 1990s with the ongoing discovery of the structure of tryptophan residues in human Rnase linking mannose via C-C bonds. By the 2000s it was understood that *C*-glycosylation may be a necessary step for ER export in mucin biosynthesis. Recently it has been possible to construct *C*-glycosylation-modified glycopeptide drugs or glycan analogs in vitro, which greatly improve biological functions. GPI-anchoring has been well understood since the complete structural elucidation in 1988 and the discovery of the initiator enzyme, Transamidase, in 1992. In 2023, the phenomenon and molecular mechanism of feedback regulation of cellular maintenance of GPI-anchored proteins were first elucidated. DPY19L, dpy-19 like C-Man transferase; Gal, d-galactose; GalNAc, *N*-acetyl-d-galactosamine; GALNT, polypeptide GalNAc transferase; Glc, d-glucose; GlcNAc, N-acetyl-d-glucosamine; GPI, glycosylphosphatidylinositol; HILIC, hydrophilic interaction liquid chromatography; OGA, O-GlcNAcase; OGT, O-GlcNAc transferase; OST, oligosaccharyltransferase; TSR, thrombospondin type 1 repeat

In recent years, glycosylation has been widely studied as a biomarker for the diagnosis and prognosis of diseases, and great progress has been made (Fig. 1). Glycans participate in various stages of tumor progression, and glycosylation provides new targets for cancer therapy through their involvement in biosynthetic pathways. Various assays to determine the structure and mechanism of action of glycosylation are also in full swing from research to clinical and scientific studies. The current focus is to utilize this knowledge and technological tools of glycosylation to develop more powerful and safer drugs that are truly applicable to patients in the clinic.

In this review, we briefly summarized the mechanisms of glycosylation, and their biological functions, and discussed the impact of glycosylation in various human diseases. We further presented the potential value of glycosylation that can be used as biomarkers for disease diagnosis and prognosis, and summarized the current assays for glycosylation, briefly discussing the advantages and limitations of each. In addition, the targeted therapies related to glycosylation, a highly promising approach for the treatment of cancer, and challenges encountered in clinical development were discussed.

## Mechanism of glycosylation

**Fig. 2:**
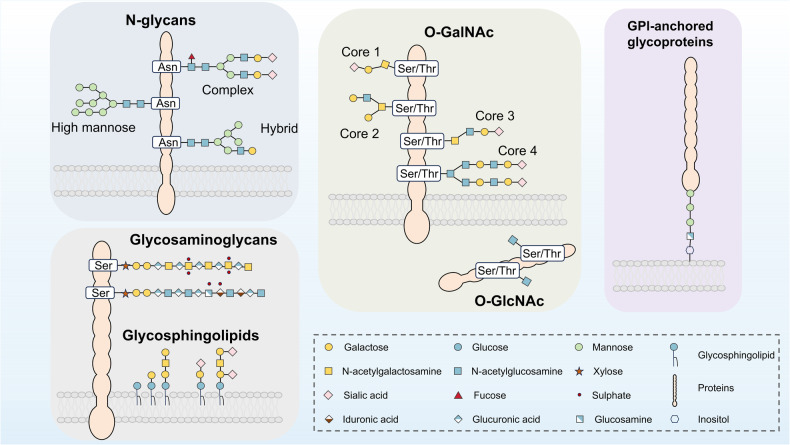
Classification of major manifestations of glycosylation on proteins. Glycosylation of proteins is capable of occurring when a saccharide is covalently attached to the polypeptide backbone via *N*-linkage to Asn or *O*-linkage to Ser/Thr. *N*-glycans that have complex, hybrid, or high mannose forms, are linked to Asn via GlcNAc. GalNAc links *O*-glycans to Ser/Thr with a variety of core structures and extensions, most of which are sialylated and fucosylated. Single GlcNAc molecules are linked to the Ser/Thr residue of intracellular proteins in the cytoplasm, mitochondria, and nucleus through the process of *O*-GlcNAc glycosylation. Certain glycoproteins known as glycosylphosphatidylinositol (GPI)-anchored proteins are also present in the plasma membrane’s outer leaflet and are connected to a phosphatidylinositol. A significant portion of the cell plasma membrane’s outer leaflet is made up of glycosphingolipids. Terminal sialic acids can be used to further modify the various series of structures that make up these ceramide-linked glycans. GalNAc, *N*-acetyl-d-galactosamine; GlcNAc, *N*-acetyl-d-glucosamine; GPI, glycosylphosphatidylinositol

Protein is essential for life and involved in all cellular processes. Proteins undergo dynamic changes and various PTMs such as phosphorylation, methylation, acetylation, and notably glycosylation.^8^ Glycosylation, being one of the known PTMs, is essential in the unfolding of various functional activities in living organisms.^9^ Glycosylation of proteins is the process of covalently binding oligosaccharides in the form of glycosides to certain amino acid residues on proteins.^10^ The amino acids and glycans are classified into four categories according to their linkage: *O*-glycosylation, *N*-glycosylation, *C*-glycosylation and GPI-anchored attachment^10,11^ (Fig. 2). Among them, *N*-glycosylation and *O*-glycosylation are the most common types and they contain most glycosylation machinery associated with the pathogenesis and progression of disease.

### *N*-glycosylation

*N*-glycosylation is a common glycosylation process facilitated by glycosyltransferases. It involves the binding of oligosaccharide *N*-acetylglucosamine (GlcNAc) to protein aspartate residues on the ER. This process results in three primary *N*-glycan subtypes, differentiated by their side chain branching into complex, mixed, and high mannose polysaccharides.^12^ *N*-glycans are conserved in the early synthesis of the ER, and their heterogeneity is seen in their subsequent processing. There is a common core structure for all *N*-glycans (asn-GlcNAc2Man3), which is even further extended by terminal glycan residues.^13^ These glycan residues, which mainly include GlcNAc, mannose, galactose, fucose and sialic acid (*N*-acetylneuraminic acid/Neu5Ac), could significantly affect the structure of *N*-glycan.

In the ER lumen, Asn-linked glycosylation (ALG) gene products assemble lipid-linked oligosaccharide precursors (Glc3Man9-GlcNAc2) onto dolpolypterpene phosphate (Dol-P) carriers. Key enzymes include GlcNAc-1-P transferase Alg7p (or GPT) with Alg13p/Alg14p uridine 5’-diphosphate-N-acetylglucosamine (UDP-GlcNAc) transferase, forming a linkage between *N*-glycan and the polypeptide chain.^14^ This assembly is followed by α-glucosidase I (GluI) and α-glucosidase II (GluII), which remove glucose residues, aiding in protein folding.^15^

The glycan is subsequently trimmed by ER mannosidase I, which removes terminal mannose residues from the branches of N-linked oligosaccharides.^16^ This structure is then recognized by lectin mannose-binding 1 (LMAN1). Matrix metalloproteinase-9 (MMP-9) is a secreted glycoprotein protease that further trims glycosylation and secretes correctly folded proteins during the passage from the ER to the Golgi apparatus. LMAN1 can act as a lectin carrier protein to mediate the efficient secretion of MMP-9, which facilitates the loading of glycosylated proteins into the coat protein complex II (COPII). COPII then transports these proteins from ER to Golgi via the ER-Golgi intermediate compartment.^17,18^ In the Golgi, various glycosidases and GTs further process the majority of *N*-glycans into complex and/or hybrid form.^19^

It was reported that aberrant expression of high mannose-type *N*-glycan structures is found in various malignancies, contributing to tumor proliferation, invasion, and migration through diverse mechanisms.^20^ However, the precise mechanism underlying the role of high mannose-based *N*-glycans in malignant tumor proliferation, immunosurveillance and tumor immunotherapy is still poorly understood and requires further investigation.

### *O*-glycosylation

*O*-glycosylation is generally an oxygen linkage of glycans to the Ser or Thr residues, followed by a gradual addition of monosaccharides. Unlike *N*-glycosylation, which generally occurs in the ER and Golgi apparatus, *O*-glycosylation occurs mainly in the nucleus and cytoplasm.^21^ The two most common types of *O*-linked glycosylation are *O*-acetylgalactosamine (*O*-GalNAc) and *O*-linked-β-D-*N*-acetylglucosamine (*O*-GlcNAc).^22^ *O*-GalNAc glycosylation is initiated by polypeptide *N*-acetylgalactosaminyl-transferase, GalNAc monosaccharides are attached to Ser or Thr residues of proteins by *O*-glycosidic bonds.^23,24^ This dense *O*-GalNAc glycosylation, also known as *O*-glycosylation mucins, is expressed in a variety of tumor types and plays a role in cell-cell interaction, and cell-matrix interactions.^25^ On the other hand, the process of *O*-GlcNAc glycosylation is primarily regulated by two enzymes, *O*-GlcNAc transferase (OGT) and O-GlcNAcase (OGA). Glucose synthesizes UDP-GlcNAc is synthesized via the hexosamine biosynthesis pathway (HBP). Subsequently, UDP-GlcNAc is attached to Ser or Thr residues via β-O bonding, thereby enhancing the extent of protein *O*-GlcNAc glycosylation in the presence of OGT.^26^ OGA hydrolyzes and removes GlcNAc from proteins and reduces protein *O*-GlcNAc glycosylation.

Dysregulated expression levels of *O*-glycan enzymes are associated with tumor development, progression, invasion, and metastasis. It is shown that mucin-type *O*-glycan biosynthesis is an important pathway of colon carcinogenesis through single-cell transcriptomic analysis.^27^ Additionally, it has been reported that *N*-acetylgalactosaminyltransferase 7 (GALNT7) is upregulated in prostate cancer and can enhance its proliferation by regulating the *O*-glycosylation of prostate cancer cells.^28^

### *C*-glycosylation and GPI anchor

*C*-glycosylation, a rare phenomenon in living organisms, involves the attachment of a mannose molecule to the tryptophan indole ring at position C via a C-C bond.^29^ The process of GPI-anchored attachment entails the binding of a GPI anchor, comprising the glycan core, to the *C*-terminus of a protein, thereby attaching it to the cell membrane. The general structure of the GPI anchor is ethanolamine, glycan core, and inositoll.^30^ GPI anchoring occurs in the ER and is a reversible modification where phospholipases can detach the anchored proteins from the cell membrane.

## Biological functions of glycosylation

### Regulation of protein functions

**Fig. 3:**
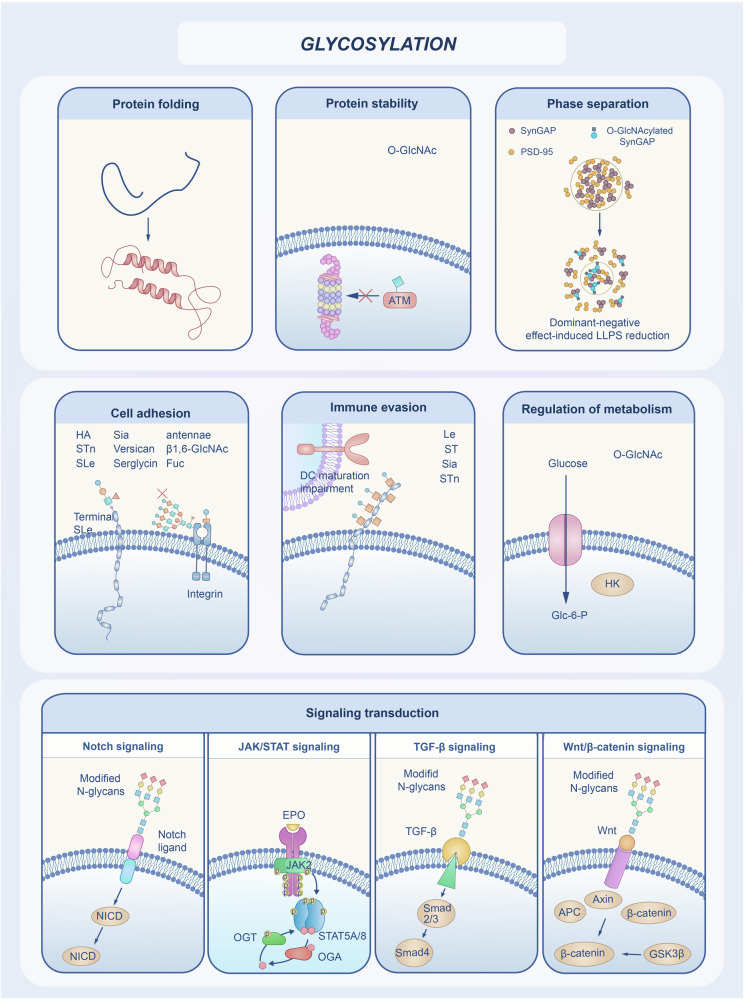
Biological functions of glycosylation. Glycosylation has a variety of biological functions that can affect protein folding and stability, for example, *O*-GlcNAcylation accelerates protein degradation in cells and decreases protein stability, it also regulates activities such as protein aggregation and phase separation. Glycosylation modulates cell-matrix interactions and promotes integrin-dependent signaling, which regulates adhesion activity. An effective immune response depends on the successful activation and maturation of dendritic cells, whereas abnormally glycosylated protein antigens impair the function of dendritic cells, allowing the cells to evade the host’s immune response. *O*-GlcNAc modifications have been found to directly regulate a variety of important biological processes within cells, such as cellular metabolism. Glycosylation can regulate enzyme activities or interact with other proteins and participate in cell signaling processes. These include Notch signaling, JAK-STAT signaling, TGF-β signaling, Wnt/β-catenin signaling pathways. GlcNAc, *N*-acetyl-d-glucosamine; LLPS, liquid-liquid phase separation; PSD, postsynaptic density; Glc-6-P, Glucose-6-phosphate; JAK-STAT, Janus kinase (JAK)-signal transducer and activator of transcription; OGT, O-GlcNAc transferase; OGA, O-GlcNAcase; TGF, transforming growth factor

Glycosylation has multiple biological functions and can affect protein folding, quality control, stability, and transportation (Fig. 3).

#### Protein folding

Protein function depends on its high-level structure, and correct protein folding plays an important role. Protein folding is the process by which a polypeptide chain folds into its natural three-dimensional structure of a biologically active protein, aligning functional groups for specificity and minimum energy. Protein folding is crucial for modulating biological activity, regulating cell growth and differentiation, and facilitating molecular transport. Misfolded proteins tend to denature and lose their structure and function, even leading to diseases such as Alzheimer’s disease, which is caused by incorrect folding of the secondary beta-structure of fibrillar beta-amyloid proteins found in the brain.^31^ Protein folding is affected by a variety of factors, including electric and magnetic fields, temperature, pH, spatial confinement and molecular crowding, and it has been found that glycosylation also plays a role in protein folding. Glycosylation is also one of the factors affecting protein folding, which on the one hand helps directly by stabilizing polypeptide structures and on the other hand can help indirectly through interactions with lectins, glycosidases, and GTs as recognition “tags”.^32^

In the ER, *N*-glycosylation directs the initial steps of protein folding and its quality control.^32,33^ *N*-glycosylation allows the newly synthesized glycoprotein to interact with the lectin-based chaperone system in the ER.^34^ In mammalian cells, calnexin, calreticulin, and related factors play a crucial role in facilitating the proper folding and oligomerization of numerous glycoproteins. They offer specialized quality control and chaperone functions tailored specifically for glycoproteins in the ER.^34^ Lectins could act as chaperones in glycoprotein folding.^34^ *O*-glycosylation stabilizes the folded protein domain and promotes protein secretion.^2^ The *O*-linked mannose structure is simpler and has recently been found to have a unique function in protein quality control that does not depend on the intricate structure of *N*-linked glycan.^35^

#### Protein stability

Glycosylation is crucial in regulating protein stability. Glycosylation can enhance the stability and solubility of proteins, thereby improving their half-life and drug properties in vivo. A recent report indicated that the *O*-GlcNAcylation of hepatocyte growth factor-regulated tyrosine kinase substrate (HGS), a critical protein in the EGFR trafficking pathway, could hasten the degradation of HGS protein in cells, thereby diminishing its stability.^36^ *O*-GlcNAcylation could stabilize PPM1K and promote dephosphorylation of BCKDHA, thereby promoting catabolism of branched-chain amino acid in hepatocellular carcinoma (HCC).^37^ CD98 (SLC3A2) is a type II glycoprotein, which is a multifunctional protein, and mutations in its *N*-glycosylation site can severely affect stability and influence migration to the plasma membrane and various cellular processes.^38^

#### Phase separation

Phase separation (or liquid-liquid phase separation, LLPS) refers to the phenomenon that biological macromolecules (such as proteins, RNA, etc.) form a temporary liquid-liquid phase separation state in the cell.^39^ Phase separation is a widespread liquid state in physical chemistry. In cells, phase separation plays a crucial role in maintaining the cellular complex structure, function and signal transmission.^40^ Recent studies have shown that *O*-glycosylation plays a crucial and multifaceted role in regulating amyloid protein aggregation and LLPS under physiological conditions.^41^ Lv et al. synthesized site-specific *O*-GlcNAc -modified SynGAP using a protein semi-synthesis strategy, and found that *O*-GlcNAcylation could modulate LLPS of SynGAP/PSD-95 complex.^42^ Furthermore, Chen et al. found that *O*-GlcNAcylation could attenuate the translation-promoting effect of YTHDF1 and YTHDF3 by inhibiting their associations with proteins involved in mRNA translation. The abundant *O*-GlcNAc modification of YTHDF1/3 enhanced its dynamic characteristics within the phase separation components and accelerated the depolymerization phase separation, low abundance *O*-GlcNAc modification of YTHDF1/3 maintained its stability in phase separation components.^43^ These results suggest that *O*-GlcNAc modification may act as a universal regulatory mechanism to regulate a variety of LLPS processes.

### Regulation of cell adhesion

Proper cell-cell and cell-extracellular matrix proteins (ECM) communications and interactions are essential to maintain healthy tissues, both structurally and functionally. These interactions are largely carried out by adhesion molecules, peripheral cell membrane proteins. Most of the proteins involved in cell adhesion are glycosylated. There are several groups of adhesions molecules on cell, including cadherin, selectin, sialic acid-binding immunoglobulin-like lectins (Siglecs), and integrin family.^44^

Different adhesion molecules apparently play different, although sometimes overlapped, roles. Cadherins, a group of transmembrane glycoproteins, are a class of Ca2+-dependent homophilic cell adhesion molecules responsible for facilitating cell-cell adhesion. There are multiple members, E-cadherin, N-cadherin, and P-cadherin in the cadherin family with E-cadherin being studied the most. Evidence suggests that glycosylation can affect the adhesion function of the family E-cadherins. E-cadherins contain a single transmembrane structural domain, a cytoplasmic structural domain (C-terminus), and an extracellular domain, and the said extracellular domain consists of five repetitive structural domains (EC 1 to EC 5).^45,46^ *O*-glycosylation of newly synthesized E-cadherin has been reported to impede cell-surface translocation, leading to decreased intercellular adhesion, which was suggested due to the impaired binding of E-cadherin to p120-catenin. In addition to phosphorylation, cytoplasmic *O*-glycosylation of E-cadherin becomes an alternative mechanism to regulate cell adhesion.^47^ The *N*-glycosylation sites of E-cadherin are located in the extracellular structural domains, with four sites clustered in the EC 4 to EC 5 region. It has been emphasized that reduced *N*-glycosylation of E-calmodulin promotes the establishment of a stable adherens-junction. However, excessive *N*-glycosylation of E-calmodulin weakens the adherens-junction.^48,49^ E-calmodulin *N*-glycosylation is mainly controlled by the GnT-III, GnT-V, and fucosyltransferase 8 (FUT8), which are involved in the regulation of cell-cell adhesion.^50^ Significant alterations in glycosylation have been observed for several cell adhesion molecules in the tumor microenvironment, predominantly cadherin, and selectins, becoming a common feature of cancer progression.

Selectins, a class of Ca2+-dependent heterophilic cell adhesion molecules, comprise three cell adhesion molecules: L-selectin expressed on leukocytes, E-selectin expressed on activated endothelial cells, and P-selectin expressed on activated platelets and endothelial cells.^4,51,52^ They could specifically recognize and adhere to specific glycosyl groups in oligosaccharide chains on the surface of other cells, and mediate the recognition and adhesion of leukocytes primarily to vascular endothelial cells or platelets, specializing in the capture of leukocytes from the bloodstream to the vessel wall.^53^ Selectins are single-transmembrane-penetrating glycoproteins with an intracellular region that binds to microfilaments via anchoring proteins. The extracellular region consists of three major structural domains: an N-terminal C lectin-like structural domain, an epidermal growth factor (EGF)-like structural domain, and a structural domain that is homologous to complement regulatory protein.^54^ Among them, the C lectin-like structural domain is the active site that recognizes specific glycosyl groups and engages in selective adhesion.^55^

Leukocytes move in a rolling fashion, mediated by selectins, by adhesion-detachment and re-adhesion-detachment with vascular endothelial cells in the vasculature at the site of inflammation.^56^ Selectins act as receptors that bind to glycoligands with structural modifications of sialyl-Lewisx(sLeX) or sialyl-Lewisa(sLea) at the end to regulate cell migration, and cell metastasis in cancer. The expression and adhesion function of sLeX on trophoblast cells and L-selectin on uterine epithelial cells mediate adhesion at the fetal-maternal interface. The key enzyme for the synthesis of sLeX is fucosyltransferase VII (FUT7), and when the expression of FUT7 was up-regulated, the synthesis of sLeX was increased, and the rate of adhesion of trophoblast cells to human uterine epithelial cells was significantly increased.^57^ There are at least 14 different Siglec receptors in human, which are predominantly found in immune cells.^58^ By binding to its ligands including sialoglycans, Siglec receptors modulate immune cell activity through recruiting SHP1 and SHP2 phosphatases.^58^ The Siglec-sialoglycan axis plays a strong role in regulating both the innate and adaptive immune responses and has been found to be strongly associated with tumor evasion which involves altered glycosylation.^59,60^ Integrins are a large family of heterodimeric alpha/beta receptors, bind to ECM so they are the molecules that “sense” the cellular environment. This binding is specific and highly regulated. The binding of integrin to specific ECM component could trigger the activation of intracellular signaling pathways, release growth factors and cytokines embedded in the ECM, and sensing the mechanic changes in the cellular environment, all of which can contribute to cell proliferation, survival, differentiation, and migration.^61–63^ Under diseased conditions, such as cancer and immunological disorders, integrins have become attractive drug targets. In fact, certain integrins are clinically validated drug targets. Changes in glycosylation of integrins observed in tumors impact the intracellular signaling and cell adhesion activity.^64^ For example, cancer patients, such as breast cancer patients, with increased GlcNAc branching on the *N*-glycans showed poor prognosis.^65^ Core 1 β1,3-galactosyltransferase (C1GALT1) modifies the *O*-glycan on integrin β1, regulates its activity, and enhances the invasiveness of HCC.^66^ β-galactoside α2,6-sialyltransferase 1 (ST6Gal-I) was identified as poor prognosis marker as hypersialylation on integrins in multiple cancers. It was found to link to increase of tumor migration in colon and ovarian cancer cells.^67,68^ Clearly glycosylation on adhesion molecules, including integrins, alters cell proliferation and function.

### Immune evasion

Immune evasion refers to the development of many mechanisms by infectious organisms (including bacteria, viruses, parasites, etc.) and cancer cells that prevent the host’s immune system to respond and trigger their elimination from the host. Glycosylation plays a critical role in immune regulation under normal and pathogenic conditions, including immune evasion by pathogenic organisms and cancer cells,^69^ which is not surprising, since most of the key molecules that are involved in immune response, both innate and adaptive, are glycosylated.^70^ The products of protein glycosylation, glycans, and the glycosylated proteins, are all well-documented in immune modulation and immune evasions by pathogens. Specifically, three major glycan-binding proteins, galectins, Siglecs, C-type lectin family (CTLF) are known to play a role in immune evasion.^71,72^ Pathogenic organisms utilize host glycans or secret their own molecules as glycan mimicry as shield to avoid immune surveillance.^73^ Cancer cells could produce abnormal amount of glycans, glycan-binding proteins, and cell surface glycosylated proteins with structural changes. The quantity and qualitative changes in glycosylation and interaction of glycosylated proteins favor tumor cells to escape the host immune response.^74^ Among the best examples, sialic acids are rich in tumor environments and have been shown to be extensively involved in a variety of cancer cells for immune suppression.^75^ Removal of sialic acids was found to be associated with tumor inhibition in glioblastoma.^76^ The changes in glycosylation, quantitively or/and qualitatively, in cell surface glycans alter the interactions between cancer cells with host glycan binding proteins such as galectins, Siglecs, and CTLF. Cell-surface glycans usually end in sialic acid, a structure that binds to a variety of endogenous receptors, among which Siglecs have been associated with immune evasion in tumor cells.^77^ Siglecs are a series of sialic acid-binding immunoglobulin-like lectins that modulate cellular functions in the innate and adaptive immune systems through glycan recognition.^58^ The extracellular portion of Siglecs contains an N-terminal carbohydrate-recognizing domain (CRD) and a C2 structural domain, and the intracellular portion contains immunoreceptor tyrosine-based inhibition motif (ITIM) or ITIM-like structures that mediate immunosuppression.^78^ Several studies have found that increased sialic acid density leads to increased involvement of inhibitory Siglecs receptors on immune cells and modulates the immune response to cancer, which has been observed in lung cancer and melanoma samples.^79^ Many cancers show an increase in sialylated glycans which may be associated with sialyltransferases, and sialic acid synthesis genes. The hypersalivation Siglecs axis may be another way for tumor cells to escape immune surveillance.^60^ Siglec-7 is involved in remodeling the sialylation status of cancer cells and thereby affecting NK cell immunoevasion.^80^ Galectins binding to LacNAc-containing glycans has an important role in regulation T cell functions.^81^ The amount and the nature of the modification of galectins all affect immune cell activity. For example, high level of Galectin-1 expression is strongly associated with low T cell infiltration to tumor tissues in head-and-neck cancers.^82^ Similarly, Gal-3 and Gal-9 were found to play a role in the downregulation of immune response in other solid tumors.^83,84^

### Modulation of signaling transduction

Glycosylation can regulate enzyme activity or its interaction with other proteins to participate in cell signaling and recognition processes.

#### Notch signaling

As a highly conserved signaling pathway, Notch signaling plays an important role in a variety of biological processes such as cell growth, development, and tissue repair.^85^ In mammals, Notch is activated by the binding of its extracellular domain to ligands (Delta and Jagged/Serrate) on the surface of apposed cells.^86^ Accumulating studies have confirmed that extracellular PTMs in the form of glycosylation are essential for the Notch signaling pathway.^87–89^ Abnormal *O*-glycosylation of Notch receptors and their ligands leading to acquired functional mutations or signaling dysregulation of genes in the Notch signaling pathway is associated with several diseases.^90^ The extracellular domain of Notch is comprised of a series of EGF repeats, which can range up to 36 in number. Studies have shown that aberrant expression of Notch-modified glycosyltransferase, including protein *O*-glucosyltransferase 1 (POGLUT1) and protein *O*-fucosyltransferase 1 (POFUT1), can be found increased in several diseases, such as oral squamous cell carcinoma.^91^ POGLUT1 functions as a protein-*O*-glucosyltransferase, facilitating the transfer of glucose to the EGF-like domains found in Notch receptors and other signaling receptors. POGLUT1 *O*-glucosylation is mainly carried out by three POGLUTs (POGLUT1, 2, and 3) that use uridine diphosphate-Glc as a substrate for modification of the EGF repetitive sequences for their *O*-glucosylation activities.^92,93^ In a family with autosomal recessive limb-girdle muscular dystrophy, a missense mutation was identified in the POGLUT1 gene. This mutation is known to induce decreased *O*-glucosyltransferase activity on Notch receptors, leading to impaired muscle development.^94^ POFUT1 is localized in the region of 20q11.21, which is heavily amplified in acute myeloid leukemia (AML).^95^ Recent findings indicate that POFUT1, the enzyme responsible for adding *O*-fucose to Notch receptors, may also play a role in quality control within the ER. Several studies have demonstrated that Fringe, a glycosyltransferase, operates within the Golgi complex to modify the glycosylation patterns of Notch receptors.^96,97^ The modulation of Jagged1-induced Notch signaling by Fringe requires the involvement of beta 4galactosyltransferase-1, which adds a galactose residue to GlcNAc beta 3Fuc. This suggests that beta 4GalT-1 may serve as a novel regulator of Notch signaling.^98^

*O*-GlcNAcylation is a common PTM, which can be divided into OGT-catalyzed intracellular form and EGF-domain specific *O*-GlcNAc transferase (EOGT)-catalyzed extracellular form. Several studies have found that *O*-GlcNAcylation is regulated by the Notch signaling pathway and is involved in disease development.^99^ OGT directly interacts with Notch1 to catalyze the *O*-GlcNAcylation of the Notch TM/ICD fragment, promoting its binding to the E3 ubiquitin ligase Itch and reducing degradation, which plays an important role in regulating mammalian neurogenesis and cognition.^100^ In addition, *O*-GlcNAcylation can also be catalyzed by EOGT, which augments Delta-like ligand-mediated Notch signaling and promotes human Adams-Oliver syndrome.^101^

Investigations conducted by multiple groups have demonstrated the crucial role of *O*-fucosylation in ligand-mediated Notch signaling. The modification of the *O*-fucose saccharide structure, induced by Fringe, influences the response of Notch to its ligands. Zhou et al. reported that Notch-dependent signaling could control myelopoiesis, and fucosylation of Notch could regulate the ligand binding activity and efficiency of Notch signaling in myeloid progenitors.^102^ *O*-fucose site mutations have been found in anaplastic large cell lymphoma as two mutations in NOTCH1 (p.T311P and p.T349P) result in deletion of the *O*-fucose site in the eighth and ninth EGF repetitive sequences in the NOTCH1 ECD.^103^ Furthermore, certain *O*-fucose molecules are elongated through the activity of β-1,3-N-acetylglucosaminyltransferases (β3GNTs) from the Fringe family. GCNT1, a glycosyltransferase, could mediate *O*-Glycosylation of the Sialomucin CD43, thereby act as an indicator of Notch signaling in T cells.^104^

#### JAK-STAT signaling

The Janus kinase (JAK)-signal transducer and activator of transcription (STAT) pathway is one of the central pathways for cellular function. A variety of cytokines and growth factors, such as hormones, interferons, and interleukins, have been identified to initiate this pathway. JAK-STAT pathway is involved in multiple processes such as immune adaptation, apoptosis, and inflammation. Deletion or mutation of JAK-STAT components is associated with a variety of diseases.^105^ The binding of cytokine to the receptor causes dimerization of the receptor, simultaneous tyrosine phosphorylation of the intracellular terminus of the receptor, and the phosphorylation of the two JAK kinase molecules that are recruited to the receptor. The phosphorylated receptor binds to the STAT protein, activating STAT phosphorylation and triggering downstream pathways.^106^

The STAT protein family includes seven members, among which STAT5 plays an important function in the regulation of growth and development and tumor immunity.^107^ *O*-GlcNAcylation promotes oncogenic transcription by enhancing STAT5 tyrosine phosphorylation and oligomerization to drive myeloid transformation.^108^ It was found that *O*-GlcNAcylation, a modification necessary for STAT5-induced transcription, occurs at Thr at the N-terminal site 92 of the STAT5 protein.^109^ Defective or hyperactive STAT5 variants of *O*-GlcNAcylation differ greatly in oncogenic potential. In Burkitt lymphoma and acquired immunodeficiency syndrome (AID)-related lymphoma, this region of the T58 locus of the c-Myc protein is a hotspot for mutation. The study could observe that *O*-GlcNAcylation of Thr at the T58 site occurs with phosphorylation modification, which also serves as evidence that *O*-GlcNAcylation regulates gene transcription signaling in tumor cells.^110^ In addition, it was identified that the JAK/STAT1 pathway could act as a regulator of glycolysis, enhanced glucose turnover was associated with abundant STAT1-*O*-GlcNAcylation in mesenchymal stem cells.^111^ The above studies demonstrate the critical role of glycosylation in the JAK-STAT signaling pathway and reveal the possible functions involved in glycosylation.

#### TGF-β signaling

TGF-β signaling plays crucial roles in the preservation of organismal integrity. TGF-β signaling governs the regulation of cell proliferation, phenotypic plasticity, metabolic adaptation, and immune surveillance in diverse cell types. Malfunctions in TGF-β signaling have the potential to disturb immune tolerance, induce inflammation, and contribute to the development of fibrosis and cancer.^112^

Both core fucosylation and sialylation primarily act as stimulatory factors in TGF-β signaling during various biological processes, including fibrosis, migration, differentiation, and immune evasion.^5^ ALG3, one of ALG members, has been to promote radiation resistance in breast cancer by regulating glycosylation of TGF-β receptor II.^113^ Kim et al. demonstrated that the use of glycosylation inhibitors, such as tunicamycin and kifunenine, or mutations in the N-linked glycosylation site of TβRII attenuated ALG3 overexpression to varying degrees, could prevente the effective transport of TβRII protein to the cell surface, thereby reducing cell sensitivity to TGF-β.^114^ Core fucosylation catalyzed by FUT8 is important for signaling receptors. Upregulated expression of FUT8 induced high levels of core fucosylation of TGF-β type I and type II receptors, facilitating TGF-β binding and downstream targets, thereby promoting cell invasiveness of breast cancer cells.^115^

Sialylation-mediated regulations on TGF-β signaling are linked with epithelial-mesenchymal transition (EMT).^116^ ST3Gal1, a key sialyltransferase, could mediate sialylation of vasorin to facilitate TGF-β1-mediated angiogenesis and progression of tumor.^117^ In addition, GalNAc-type *O*-glycosylation is initiated by polypeptide *N*-acetylgalactosaminyltransferases, is involved in TGF-β signaling regulation. Inhibition of breast cancer cell migration and invasion, specifically through the EMT process, could be achieved by targeting ppGalNAc-T4-catalyzed TGF-β receptor *O*-GalNAcylation. This suggests that focusing on ppGalNAc-T4 may hold promise as a therapeutic strategy for breast cancer.^118^ Together, these studies suggested the regulatory role of glycosylation in TGF-β signaling, more investigations are needed to better understand the process and its implications.

#### Wnt/β-catenin signaling

The Wnt signaling pathway plays a pivotal role in regulating cell-fate determination, cell migration, cell polarity, neural patterning, and organogenesis during embryonic development.^119^ Abnormal Wnt signaling can lead to developmental diseases or cancer. Wnt is a class of secreted protein molecules with glycosylation and fatty acidification modification. To date, 19 Wnt proteins have been identified in humans. Wnt pathway is commonly divided into β-catenin-dependent (canonical) and independent (non-canonical) signaling.^120^ Among the mechanisms that have been documented to affect Wnt/β-catenin activity, modification of *N*-glycans by L-fucose is the recently understood. Hong et al. revealed that increased α (1-3)-fucosylation of GlcNAc in the Galβ (1-4)-GlcNAc sequences of complex *N*-glycans could suppress the activation of Wnt/β-catenin signaling through elevates the endocytosis of lipid-raft-localized low-density lipoprotein receptor-related protein 6 (LRP6).^121^ Conversely, Wnt suppression induced by cell-surface α (1-3)-fucosylation can be rescued by addition of free fucose.^121^ B3GnT2, a member of the β1,3-N-acetylglucosaminyltransferase (B3GnT) family, could promote the extension of polylactosamine chains on multiple *N*-glycans on LRP6, thereby enhancing LRP6 transport to the plasma membrane and promoting Wnt/β-catenin signaling.^122^

In addition, β1,4-Galactosyltransferase V is engaged in embryogenesis and plays a crucial role in catalyzing the attachment of galactose to GlcNAcβ1-4Man residues located on *N*-glycans. This enzymatic process holds significance in regulating cell stemness through glycosylation mechanisms, leading to the stabilization of Frizzled-1 and activation of the Wnt/β-catenin signaling pathway specifically in breast cancer.^123^ Overexpression of the polypeptide *N*-acetylgalactosamine-transferase 1 could activate the Wnt/β-catenin signaling through modulating CD44 *O*-glycosylation in gastric cancer.^124^ Further validation of the regulatory mechanism of glycosylation will contribute to a better understanding of the function of Wnt/β-catenin signaling.

### Regulation of metabolism

Recently, accumulating studies have demonstrated the importance of protein glycosylation in regulating lipid metabolism.^125^ *N*-glycosylation plays a regulatory role in proteins involved in lipid synthesis, packaging, and the clearance of lipoproteins. *N*-glycosylation in humans reduces low-density lipoprotein by enhancing low density lipoprotein (LDL) receptor expression, suggesting that *N*-glycosylation could act as a vital regulator of LDL metabolism.^126^ In addition, the specific glycan patterns of high-density lipoprotein (HDL)-associated ApoE are intricately involved in the activity and functionality of HDL. The enzyme galNAc-T2, encoded by GALNT2, plays a crucial role in catalyzing the attachment of GalNAc in *O*-glycosylation processes. Genetic studies have demonstrated that the variants in GALNT2 could impact HDL and triglyceride levels in humans.^127^

In terms of glutamine metabolism, a study on pancreatic ductal adenocarcinoma revealed that the cancer cells are highly dependent on a specific glutamine catabolic pathway, and malate dehydrogenase (MDH1) is a key enzyme in this pathway.^128^ Zhu et al. found that Ser189 was the glycosylation site of MDH1, and its *O*-GlcNAc glycosylation could promote Gln catabolism. Moreover, Ser189 *O*-GlcNAc could act as a “molecular glue” to stabilize the substrate binding pocket on the MDH1 monomer, improve substrate binding and stability, and ultimately promote the enzymatic activity of MDH1.^129^ Further determination of the mechanism by which protein glycosylation modifies metabolic reprogramming will open up novel insights for therapeutic intervention in metabolic diseases and tumors.

## Abnormal glycosylation in human diseases

**Fig. 4:**
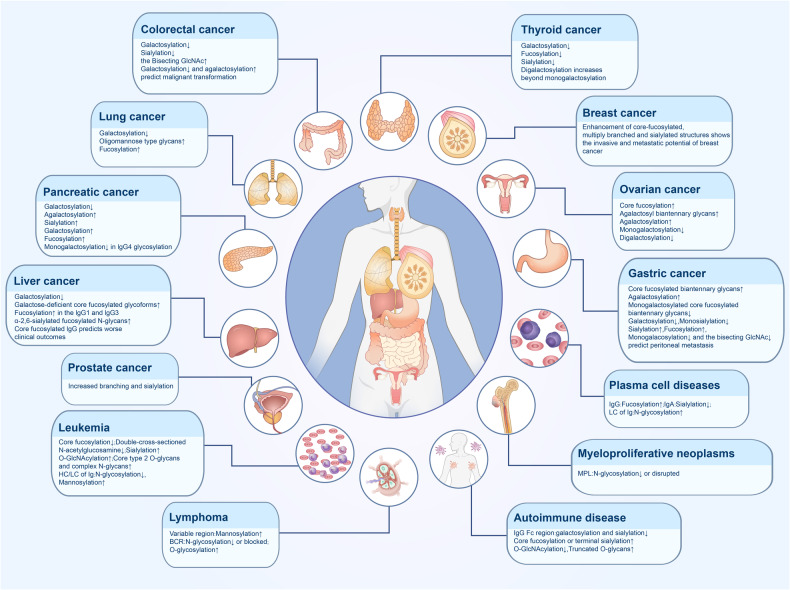
Altered glycosylation in human disease. Glycosylation is widely present in organisms, and aberrant glycosylation is closely associated with the development of a variety of diseases. Altered glycosylation present in solid tumors, hematological malignancies, and autoimmune diseases are summarized in the figure

Glycans, together with proteins, nucleic acids and lipids, are important biomolecules in the human body. Protein glycosylation is widespread in living organisms and involves various processes such as cellular immunity, protein translation regulation, and signaling pathways. Abnormal glycosylation is associated with the onset and progression of various diseases^130^ (Fig. 4).

### Glycosylation in hematologic malignancies

Hematological malignancies are a group of diseases with high heterogeneity in molecular and phenotypical characteristics. Increasing evidence suggests that protein glycosylation is involved in the pathogenesis and development of hematological malignancies, including lymphoma, leukemia, and plasma diseases.

#### Leukemia

##### Acute myeloid leukemia

Acute myeloid leukemia (AML) is a highly diverse malignancy originating from hematopoietic stem and progenitor cells.^131^ Fms-like tyrosine kinase 3 (FLT3), a receptor tyrosine kinase of type III, plays a critical role in regulating the growth and maturation of hematopoietic cells, is one of the commonly mutated genes in AML.^132^ Mutations in the FLT3 gene are detected in about 30% of adult AML patients, and the mutations are mainly in the FLT3-internal tandem duplication (FLT3-ITD) and FLT3-tyrosine kinase domain (FLT3-TKD), of which FLT3-ITD mutation is more common and suggests a poorer prognosis.^133^ FLT3 is synthesized in the ER as a 130 KD precursor of glycosylation-deficient or mannose-rich material, is transferred to the Golgi apparatus and then continues glycosylation to complete the complex 150 KD structure before transferring to the cell surface.^134,135^ If the progression of FLT3 in the Golgi is disrupted, FLT3 protein carrying immature glycosylation is then accumulated in the ER. This promotes apoptosis or initiates signaling pathways that are distinct from normal glycosylation.^136^ It has been illustrated that knockdown of FUT8 leads to the deletion of core fucosylation of FLT3, which results in sustained ligand-independent and autonomous activation of its downstream pathway and unlimited proliferation of tumor cells.^137^ 2-Deoxy-D-glucose (2DG) could induce cell death by inhibiting the *N*-glycosylation of FLT3 in FLT3-ITD-mutated AML, decreasing the cell surface expression of FTL3-ITD. 2DG is also effective in c-KIT-mutated AML cells. In the latter, *O*-glycosylation of Sp1 was modulated and c-KIT expression was decreased, triggering ER stress and activation of the unfolded protein response, which induced apoptosis.^138–140^ Tyrosine kinase inhibitors (TKIs) increase the surface level of FLT3 as well as FLT3-ITD and FLT3-D835Y mutants by upregulating their glycosylation.^141^ Statins such as lovastatin can reduce mutant FLT3 kinase activity by blocking complicated glycosylation at the receptor. This in turn contributes to changes in positioning and signaling, leading to cell apoptosis.^136,142^ Therefore, TKI combined with FLT3 immunotherapy, which attenuates the level of glycosylation, may benefit AML patients.

In addition, the interaction between AML and the bone marrow microenvironment is crucial in disease progression.^143^ In MDS/AML patients, a significant down-regulation of double-cross-sectioned GlcNAc structures was observed in bone marrow stromal cells, whereas enhanced expression of MCAM, a marker of bone marrow niche, further activates the ERK signaling pathway and promotes bone marrow cell growth.^144^ The membrane scaffold CD82 in the microenvironment undergoes *N*-glycosylation, and inhibition of its glycosylation increases the molecular accumulation of N-cadherin, promotes bone marrow homing of AML cells, and affects AML cell migration in vivo.^145^

Studies have revealed that leukemia cells expressing CD43 are enriched with sialic acid glycosyl groups on their surface, while CD43 is a heavily *O*-glycosylated protein, and the glycosylation status of the CD43 protein was associated with cytotoxic T cell (CTL)-mediated cytolysis.^146^ Sialic acid residue epitopes on CD43 have been studied as targets for bispecific T cell-engaging antibodies, which have been shown to specifically induce CTL cells and mediate cytotoxicity in both in vivo and in vitro studies, providing evidence for the therapeutic potential of targeting the sialic acid residue epitope of CD43.^147^ The role of sialic acid modification on CD43 demonstrates the relevance of glycosylation in AML pathophysiology. At present, there are more emerging studies that have taken a deeper dive at glycan characterization. It can be concluded that glycosylation plays a major part in the development and treatment of leukemia. However, more comprehensive studies on their mechanisms are needed in order to provide a stronger rational for the development of new therapies for AML and new perspectives for targeted therapy.

##### Acute lymphoblastic leukemia

Acute lymphoblastic leukemia (ALL) is characterized by the uncontrolled growth and infiltration of immature lymphocytes in the bone marrow, peripheral blood, and other organs including B-ALL and T-ALL. High levels of C-Myc expression promote leukemia cells to enhance self-renewal and be more insensitive to chemotherapy-induced differentiation.^148^ *O*-GlcNAcylation, plays a pivotal role in modulating the activity of nuclear proteins involved in gene expression, signal transduction, and cell growth.^149^ High expression of *O*-GlcNAcylation of c-Myc promotes rapid proliferation of pre-B cells. Evidence of decreased pre-B cell number was also observed in mice administered *O*-GlcNAc inhibitor.^150^ Oliveira et al. found that B-cell precursor ALL (BCP-ALL) cells with mixed lineage leukemia gene rearrangement exhibited increased core type 2 *O*-glycans and complex *N*-glycans, alongside significant changes in sialic acid and fucose glycosylation.^151^

The role of glycosylation is also captured in T-ALL. CD95 (Fas/Apo-1), a type I transmembrane protein encoded by the Fas gene that binds to CD95L and induces apoptosis, through which virus-infected cells, damaged cells and cancer cells are eliminated.^152^ In a study treating cells with interferon-gamma, it was found that the high molecular weight form CD95, most of which is caused by *N*-linked glycosylation.^153^ SHIP-1 (SH2 (Src homology 2)-containing inositol 5’-phosphatase-1) is a negative regulator of the immune response, partially located in the ER, and promotes the glycosylation of CD95, resulting in its inability to oligomerize, leading to the formation of the death-inducing signaling complex and impaired downstream apoptotic cascades.^154^

##### Chronic lymphocytic leukemia

Chronic lymphocytic leukemia (CLL) is a mature B-lymphocyte clonal proliferative neoplasm, characterized by lymphocyte aggregation in the peripheral blood, bone marrow, spleen and lymph nodes.^155^ The B cell receptor (BCR) is an immunoglobulin expressed on the membrane surface of B lymphocytes during B lymphocyte development, and is a specific receptor for B lymphocytes to recognize and bind antigens. The structure of the immunoglobulin heavy chain (IGH) is critical for the function of BCR.^156^ After the development and maturation of B lymphocytes, the IGH is stimulated by antigen and undergoes somatic high-frequency mutation (SHM), which enhances the affinity of the BCR for the antigen. In CLL cells, the IGH of the BCR can be characteristically altered.^157^

CD79 is a heterodimer molecule involved in signal transduction as a component of BCR. CD79 has two subunits, CD79a and CD79b (Igα and Igβ, respectively), and is highly expressed in CLL cells.^158^ A study showed that low levels of BCR expression in CLL cells was due to impaired glycosylation and folding of mu and CD79a.^159^ SHM frequently leads to the creation and/or disruption of *N*-glycosylation sites within the variable structural domains of the Ig H and L chains, which appears to be related to the natural history of CLL.^160^ In addition, the heavy chain constant region of the BCR in CLL cells carries a high level of mannosylation modification, which could relate to a more aggressive CLL.^161^

#### Lymphoma

##### Follicular lymphoma

Lymphoma is one of the first hematologic malignancies to be identified.^162^ Non-Hodgkin lymphoma (NHL) is the most prevalent hematologic malignancy globally, responsible for approximately 3% of cancer cases and fatalities. Follicular lymphoma (FL) is a class of NHL originating from follicle-centered B-cells, with a typical immunophenotype of CD5-CD10 + CD19+ with t(14;18) (q32; q21), and whose BCR undergoes SHM to produce a high degree of tumor heterogeneity.^163,164^ FL cells undergo persistent somatic hypermutation in the variable region gene of their immunoglobulins. DNA sequences encoding Asn-X-Ser/Thr (*N*-glycosylation site) were introduced by sustained somatic hypermutation in the variable region.^165,166^ Following contact with calcium-dependent lectins exposed to tissue macrophages, the unique additional oligosaccharide activates the BCR signaling pathway. That route seemed to be crucial for tumor growth and proliferation.^167^

*N*-glycosylation sites are introduced into the variable region of the BCR in FL and exhibit a high mannose type. Binding of these glycosylated groups to mannose-specific lectins triggers sustained BCR signaling activation, which is critical for tumor cell survival and proliferation.^161^ The introduction of *N*-glycosylation sites by SHM can occur in a variety of B-cell diseases, but has the highest prevalence in FL. Most of the neo-glycosylation sites are located in the complementary decision region in the variable region and less frequently in non-functional V(H) sequences.^166^ High throughput sequencing analysis revealed that sustained SHM could result in subclones with different amino acid compositions in different disease events, but a large majority of the resulting DNA sequences still encode an *N*-glycosylation site.^167^ In addition, a recurrent type of *N*-glycosylation, NX(S/T), was identified in mutations in the variable structural domain in FL.^168^ This study suggested that the opportunistic interactions occurred at BCR and cells containing mannose-binding lectins may contribute to the pathogenesis of FL.^169^

Studies addressing differences in *N*-glycosylation patterns between FL subgroups with and without t(14;18) found a significant difference in the frequency of newly acquired *N*-glycosylation sites in t(14;18)-positive and t(14;18)-negative FL stage III/IV patients, which was not observed in FL stage I/II patients.^170^ In contrast, newly acquired *N*-glycosylation sites were significantly reduced in t(14;18)-negative advanced (stage III/IV) FL, suggesting that signaling in addition to the current BCR pathway. It has been found that *N*-glycosylation site-negative FL cells can also expand and may be more dependent on the energy metabolism pathway than the BCR signaling.^171^

##### Diffuse large B-cell lymphoma

Diffuse large B-cell lymphoma (DLBCL) is the most prevalent type of NHL, which derived from mature B cells.^172^ Glycosylation in different proteins can regulate the development of DLBCL from multiple pathways. BCR signaling pathway plays an important role in the development of DLBCL. Inhibition of BCR glycosylation by oligosaccharyltransferase-B reduces BCR clustering and internalization, thereby attenuating PI3K and NF-κB activation.^173^ This finding suggests that agents against the oligosaccharyltransferase-B glycosylation pathway may be useful for the treatment of DLBCL.

CD45 is classified as a type 1 transmembrane protein tyrosine phosphatase, a glycoprotein of 180-220kDa.^174^ CD45 consists of two cytoplasmic domains, a transmembrane domain and an extracellular structural domain, and mature CD45 is highly *O*- and *N*-glycosylated.^175^ Gal-3 is a soluble beta-galactoside-binding protein that is widely distributed in various tissues and organs.^176^ Gal-3 binds to a variety of ligands on the cell surface and in the extracellular matrix, and plays an important role in apoptosis, adhesion, proliferation, migration, inflammatory response, and immune response. Gal-3 is overexpressed in DLBCL and binds mainly to CD45, which is regulated by C2GnT-1 glycosyltransferase.^177^ The activity of tyrosine phosphatase is regulated by the binding of gal-3 to CD45. Removal of gal-3 from CD45 on the cell surface with the multivalent glycan inhibitor GCS-100 increased the sensitivity of DLBCL cells to chemotherapeutic agents.^178^ This sheds light on the importance of specific glycosylation of CD45 in regulating gal-3 pathway signaling and implication in DLBCL chemotherapy treatment.

Tumor immune escape mediated by the programmed cell death molecule 1 (PD-1)/PD-L1 signaling pathway is one of the important mechanisms of tumorigenesis and progression.^179^ PD-1 is mainly expressed on the surface of activated T cells, and its ligand PD-L1 is mainly exposed on the surface of tumor cells. The binding of PD-1 to its ligand PD-L1 affects the downstream signaling pathway through dephosphorylation of key molecules and inhibits T-cell receptor/CD28 signaling. Thus, it acts as a negative immune response regulator, leading to immune escapement of neoplastic cells.^180,181^ Immune checkpoint inhibitor drugs, anti-PD-1 or anti-PD-L1 antibodies, have been developed and found to be efficacious in treating a variety of human cancers.^182^ Glycosyltransferase 1 domain-containing 1 (GLT1D1) was found to be highly up-regulated in early relapsed DLBCL. GLT1D1 transfers *N*-linked glycans to PD-L1, which was found to promote the immunosuppressive function of glycosylated PD-L1 and attenuates the cytotoxic function of T cells against lymphoma cells.^183^ Thus, inhibition of GLT1D1 activity may be a new therapeutic direction for DLBCL.

#### Plasma cell diseases

##### Multiple myeloma

Plasma cell disease is a heterogeneous group of diseases of plasma cell origin caused by the over-proliferation of clonal plasma cells or that produce immunoglobulins.^184^ Monoclonal immunoglobulins or their fragments (called M-proteins) are emitted in most cases in serum or urine, with some end-organ damage. The synthesis and secretion of excess, structurally homogeneous M-protein is a common feature of plasma cell disease.^185^ IgG is the most abundant in serum immunoglobulins. The decrease or deficiency of IgG makes individuals susceptible to repetitive infections, demonstrating its role in immune defense.^186^ Glycosylation is important for the functional realization of IgG. It was previously reported that IgG from selected myeloma patients had a high degree of fucosylation in the Fab fraction.^187^ Multiple myeloma (MM) patients with bone disease showed less galactose on IgG than patients without bone disease. Moreover, less sialic acid on IgG was found in MM patients, compared to healthy control.^188^ Fucosylation glycosylation-modified IgG could play a role in myeloma development. Compared to IgG, IgA subtypes in MM are relatively rare. The level of IgA could have value, but more studies are needed.^189^ A study that collected sera from 35 patients with monoclonal IgA, isolated and purified IgA, and found that patients with monoclonal gammopathy of undetermined significance (MGUS) had less sialylated IgA than healthy volunteers.^190^

In addition to exploring the mechanisms of plasma cell disease development, glycosylation can also be potentially valuable in its diagnosis and staging. For example, serum *N*-glycoprotein changes were significant in the pathologic findings of IgD-MM. Different *N*-glycans levels correspond to different peaks and can be used in the differential diagnosis of IgD-MM and light chain (LC)-MM.^191^

##### Light-chain amyloidosis

Light-chain amyloidosis (AL) is caused by the misfolding of immunoglobulin LCs. A study using cryo-electron microscopy to demonstrate the structure of λ1-AL amyloid fibrils showed that *N*-glycosylation acts as a key regulator in fibrin misfolding.^192^ A study using MASS-FIX, an immunoenrichment-based matrix-assisted laser desorption ionization time-of-flight mass spectrometry, confirmed the LC *N*-glycosylation in 5% of MASS-FIX-positive patients with clinically significant rare monoclonal diseases including AL amyloidosis and cold agglutinin disease.^193^ In addition, Nevone et al. revealed the specific sequence and spatial pattern of *N*-glycosylation in amyloidogenic κ LCs, where the majority of *N*-glycosylation sites are located in framework region 3, specifically inside the E chain.^194^ Monoclonal LC *N*-glycosylation is a detrimental element for progression to AL amyloidosis, myeloma, and other plasma cell diseases, and it is hoped that these findings will yield an earlier diagnosis and minimize morbidity and mortality.^195^ Single-cell RNA sequencing presented the upregulation of transcriptional program of glycosylation-modified, tumor-associated plasma cells in AL, which may provide a new direction for understanding plasma cell development and transcriptional reorganization.^196^ Although important links between glycosylation and plasma cell disease have been identified, the precise functions and molecular mechanisms involved still require more detailed studies to be fully elucidated.

#### Myeloproliferative neoplasms

Typical myeloproliferative neoplasms (MPN) are classified as chronic myeloid leukemia, polycythaemia vera, primary thrombocytosis, and primary myelofibrosis.^197^ JAK2(V617F) point mutation is strongly associated with MPN. Cell surface type I cytokine receptors such as erythropoietin receptor, granulocyte colony-stimulating factor receptor (G-CSFR), and thrombopoietin receptor are homodimers. When cytokines bind to their receptors, JAK2 forms a dimer and phosphorylates each other, which in turn activates STAT5 to exert transcription factor activity and ultimately causes the generation, proliferation and differentiation of the corresponding hematopoietic progenitor cells.^198^ JAK2, myeloproliferative leukemia protein (MPL), and CALR were found in close correlation with the development, progression and regression of MPN, and all of them were involved in glycosylation, and the activation of JAK-STAT may be the central link in the pathogenesis of MPN.^199^ Mutations in the CALR gene encoding the ER-resident chaperone protein calreticulin are associated with most MPN, and deletion and insertion of its exon 9 leads to the 21/12 transition.^200^ The mutated calreticulin activates MPL by binding to the *N*-glycosylation site of MPL through its new C-terminus, particularly the two Asn residues of *N*-117 and *N*-178, which in turn induces ligand-independent activation of the JAK2-STAT/ PI3K and MAPK pathways.^201^ In mutant CALR-transformed cells, genes that have undergone *N*-glycosylation are depleted to varying degrees. Chemical inhibition of *N*-glycosylation expression on the surface of MPL cells impairs the growth of mutant CALR-transformed cells.^202^

Granulocyte colony-stimulating factor (G-CSF) is a polypeptide chain of granulocyte growth factor that mobilizes a variety of bone marrow precursor cells by binding to the G-CSFR to promote the proliferation, differentiation and migration of granulocytes and enhance the function of mature granulocytes.^203^ CSF3R is the gene encoding G-CSFR, and acquired mutations in CSF3R have been associated with malignant events.^204^ Mutations in CSF3R are common in patients with chronic neutrophilic leukemia (CNL) or atypical CML.^205^ CSF3R T618I mutation is a point mutation in the proximal extra-membrane structural region, as T618I is located at the *O*-glycosylation site, and glycosylation changes at this site are associated with tumorigenesis.^206^ There is also a rarer point mutation at N610 of CSF3R, as the Asn residue at N610 is N-linked.^207^ The mutation disrupts the *N*-glycan as part of the consensus glycosylation motif, losing its role in folding, transport and thermodynamic stabilization of the protein.^208^ These N610 mutants allow CSF3R to activate independently of its ligand G-CSF, promoting tumor cell expansion through the JAK-STAT signaling, with potent oncogenicity. Kinase inhibitors can prevent the growth of cells possessing this mutation.^203,208^ Point mutations in CSF3R T618I and CSF3R N610 hyperactivate CSF3R, resulting in cytokine-independent growth of leukemia cells.^208^ The above studies revealed the critical function of *N*-glycosylation in maintaining the normal function of CSF3R, and provide a basis for conducting studies on the regulation of glycans in receptors.

### Glycosylation in solid malignancies

Glycosylation changes associated with cancer were first identified more than 50 years ago.^209^ Today, aberrant alterations in glycosylation are widely recognized in various cancers. These alterations include truncated *O*-glycans, altered *N*-glycan branching, and the incorporation of aberrant glycan chains.^210,211^ Tumor cells may experience aberrant glycosylation due to several factors, firstly, including the aberrant expression of GTs and hydrolases, changes to the peptide backbone or glycan chain backbone, and variability in substrates and donor availability.^212–215^ Recognizing abnormal glycosylation as a hallmark of cancer has been pivotal. In-depth investigation into the mechanisms of glycosylation in cancer development is now a critical aspect of tumor biology research.^67,216^

#### Respiratory neoplasms

Lung cancer is a prevalent malignant tumor that is associated with high morbidity and mortality rates worldwide.^217^ The role of glycosylation in lung cancer has been an area of significant research interest, and several important findings have been made in recent years. Studies have shown that lung cancer cells often exhibit altered glycosylation patterns compared to normal lung cells.^218,219^ These changes include variations in *N*-linked and *O*-linked glycosylation, which can affect the behavior of cancer cells, including their growth, invasion, and metastasis.^220^ For instance, changes in the glycosylation of adhesion molecules can influence the ability of cancer cells to detach from the primary tumor and spread to other parts of the body.^221^ Importantly, aberrant glycosylation in lung cancer can help the tumor evade immune detection and induce resistance to immunotherapy including ICIs. Glycosylation of PD-L1 is a major mechanism that induces its stabilization, reduces the detection by PD-L1 antibody and consequently results in immune escape.^222^ Removing the *N*-linked glycosylation significantly enhanced anti-PD-1/PD-L1 therapy efficacy.^223^ In addition, changes in glycosylation patterns in lung cancer cells also have potential as biomarkers for early diagnosis and prognosis. Certain glycan structures have been identified that are uniquely present or altered in lung cancer, which could aid in detecting the disease early and predicting patient outcomes.^224,225^ Ongoing studies continue to uncover more about how glycosylation influences lung cancer at the molecular level, which may shed light on new therapeutical targets and strategies.

#### Gastrointestinal tumor

As reported previously, the digestive system cancers often exhibit altered glycosylation patterns, which affect cell signaling, adhesion, and invasion, playing a crucial role in cancer development and progression.^226–228^ For instance, increased sialylation and fucosylation of glycoproteins and glycolipids on cancer cells can enhance cell ability to detach, invade, and form metastases in colorectal cancer.^229,230^ Modified glycan structures on the surface of cancer cells can impair the immune system’s ability to recognize and target them, facilitating tumor growth and survival.^228,231^ Glycosylation in digestive system cancers also affects the tumor microenvironment, affecting tumor growth and response to therapy.^232^ The aberrant glycosylation pathways in digestive system cancers present new targets for therapeutic intervention.

Moreover, sialylation is mediated by sialyltransferases, and increased overall sialylation, particularly ST6Gal-I-mediated α2,6-linked sialylation is strongly associated with gastrointestinal tract cancers, as well as being a marker of poor prognosis.^233,234^ Further cellular and animal experiments have also shown that ST6Gal-I is associated with invasiveness and metastasis in gastrointestinal tumor.^68,235^ It was found that most hemagglutinins showed diminished binding to β-galactosides ending in α 2,6-sialic acid.^236^ ST6Gal-I adds an α2,6-sialic acid to the terminal galactose of the *N*-glycan, a modification that prevents binding of hemagglutinin to β-galactoside and resists galectin-mediated apoptosis.^237^

sLeX is a ligand for selectin, a vascular cell adhesion molecule belonging to the family of C-type lectins. The production of the selectin-recognized sLeX and sLea tetrasaccharides induced by increased sialylation of the outer chains of the *N*-glycan branches may also be one of the mechanisms that enhance the progression of the cancer process. The high expression of sLeX and sLea in tumor cells correlates with a poor prognosis in patients with gastrointestinal cancers.^238^ sLeX interacts with selectin to induce cancer cell adhesion to platelets and arrest on endothelial cells, regulating tumor cell metastasis in colorectal carcinoma.^239^ sLea, or carbohydrate antigen19-9 (CA19-9), is the commonly used biomarker for pancreatic cancer, and high expression is also detected in gastrointestinal tumors.^240^ The CA19-9 locus has now become one of the research targets for cancer therapy, and therapeutic strategies such as antibodies and vaccines, and CA19-9-guided nanoparticles are emerging.^241^

#### Reproductive cancers

The reproductive cancers, including cervical, ovarian, and endometrial cancers in women, and prostate cancer in men, exhibit varied incidence rates. The role of glycosylation in cancers of the reproductive system has been the subject of significant research, revealing critical insights into how these complex biochemical modifications influence the development, and treatment of these cancers.^242,243^

##### Endometrial cancer

Altered glycosylation patterns in endometrial cancer have been identified, with changes in *N*-linked and *O*-linked glycosylation affecting tumor growth and the potential to metastasize.^244,245^ These alterations in glycosylation patterns can also impact the immune system’s ability to recognize and respond to the tumor.^246^ Changes in glycosylation patterns in reproductive system cancers offer potential as biomarkers. For instance, certain glycosylation patterns in ovarian and endometrial cancers may aid in early diagnosis, monitor disease progression, and predict response to therapy.^247–249^ Targeting specific glycosylation pathways and enzymes involved in glycosylation has shown promise in preclinical models, suggesting potential therapeutic strategies. It is also worth noticing that the altered glycosylation in these cancers not only contributes to tumor progression but also impacts the fertility and overall quality of life of patients, making it a critical area of research for patient-centric therapies.^250^

##### Ovarian cancer

Aberrant glycosylation has been observed in ovarian cancer, particularly in the form of altered sialylation and fucosylation patterns. These changes in glycosylation affect cell adhesion, migration, and invasion, which are critical for tumor metastasis.^251,252^

##### Prostate cancer

In prostate cancer, changes in glycosylation, particularly in the forms of complex *N*-glycans, have been linked to tumor progression and metastasis. Specific glycan structures, such as increased branching and sialylation, have been associated with more aggressive diseases.^28,253,254^

#### Breast cancer

Abnormal glycosylation in breast cancer impacts cell signaling, adhesion, and immune recognition, contributing to the cancer’s aggressiveness.^6,255^ Moreover, aberrant glycosylation enables breast cancer cells to evade immune detection, facilitating tumor progression.^181,256^ Current biomarkers for breast cancer prognosis and treatment still lack applicability and precision. Glycosylation patterns in breast cancer are potential biomarkers for early diagnosis, prognosis, and treatment monitoring, with unique glycan structures identified in breast cancer cells.^257^ Targeted therapies aiming at these altered glycosylation pathways, such as inhibiting specific glycosylation enzymes, show promise in reducing tumor growth and enhancing treatment efficacy. Furthermore, research suggests glycosylation characteristics might vary among breast cancer subtypes, like TNBC indicating the possibility of subtype-specific treatment approaches.^258,259^

Basement membrane degradation and myoepithelial cell reduction are key features of breast cancer progression, and glycosylation plays a role in disrupting cell-cell adhesion and cell polarity, driving breast cancer progression.^260,261^ E-calmodulin is a cell surface glycoprotein that is essential in mammary cell development and epithelial cell dissemination. When E-calmodulin undergoes down-regulation of *O*-mannosylation and an increase in N-branched complex glycans, its signaling is aberrant and cell cycle progression is dysregulated.^262^ Breast cancer cells present upregulation of glycolysis, prompting some glucose to enter the HBP, increasing the level of *O*-GlcNAc and enhancing the expression of OGT, regulating cell metabolism, promoting tumor cell growth and accelerating cancer progression.^263^ Studies have shown that OGT can be inhibited by targeting the oncogenic transcription factor FoxM1, which is a potential new therapeutic target for breast cancer.^264^ These insights affirm glycosylation’s pivotal role in breast cancer pathophysiology.

The figure of glycosylation is very common in breast cancer cell metastasis and invasion. Matteo Rossi et al. found that deletion of phosphoglycerate dehydrogenase (PHGDH) in breast cancer patients promotes metastatic spread of tumor cells. Low expression of PHGDH loses its interaction with phosphofructokinase, which activates the hexosamine-sialic acid pathway, and increases sialylation of integrin α_v_β_3_, thus facilitating cancer cell migration and invasion.^265^ Paweł et al. found that podoplanin (PDPN) from a subpopulation of breast cancer tumor-associated macrophages (TAMs) promotes cancer lymphangiogenesis and distant metastasis through attachment to lymphatic endothelial cells (LECs).^266^ And this process is predicated on the presence of a large amount of *O*-glycosylation in the extracellular structural domain of PDPN. Klára et al. found metastatic site specificity of *N*-glycosylation in breast cancer metastasis. While high-mannose glycans were most frequently elevated in breast cancer metastases, it was the increase in core fucosylation that was more pronounced in bone metastases.^267^ This suggests the importance of glycosylation as a diagnostic marker for metastatic breast cancer.

Inhibition of glycosylation has potential in tumor immunosuppression.^258^ Li et al. found that targeting glycosylated PD-L1 and blocking PD-L1/PD-1 interactions promoted PD-L1 internalization and degradation, and was able to eradicate TNBC cells.^181^ Monoclonal antibodies targeting glycosylation have become another direction of immune checkpoint therapy. Targeting glycosylation may be a strategy to combat breast cancer drug resistance. Researchers have found that upregulation of dolichyl-phosphate N-acetylglucosaminyltransferase (DPAGT1) maintains high levels of HER2 shedding, which produces trastuzumab resistance in breast cancer. And this process relies on DPAGT1 to induce *N*-glycosylation levels of the shedding enzyme, ADAM metallopeptidase domain 10 (ADAM10).^268^ Blocking the glycosylation level may be a new direction to combat trastuzumab resistance. Naoomi et al. found that by inhibiting the expression of ribophorin II (RPN2), the level of *N*-glycosylation of the tetraspanin CD63 could be reduced, leading to the localization of multidrug resistance protein 1 (MDR1) to be moved out of the cell surface, and decreasing drug resistance in malignant breast cancer cells.^269^ Glycosylation plays a variety of roles in the process of breast cancer occurrence, progression and migration, and also provides a variety of new target directions for tumor therapy, which still requires in-depth study of its mechanism to explore its endless clinical potential in the future.

#### Central nervous system tumors

Aberrant glycosylation in central nervous system (CNS) tumors has been linked to tumor growth, invasion, and metastasis.^270^ For instance, alterations in the glycosylation of cell surface proteins can influence the way cancer cells interact with their environment, facilitating invasion into surrounding brain tissue.^271^ Glycosylation can significantly impact the tumor microenvironment in the CNS. This includes altering interactions with immune cells, impacting the blood-brain barrier, and influencing the response to therapies.^272^ Glycosylation in CNS tumors also affects how these tumors interact with normal neural cells. For instance, altered glycosylation patterns in CNS tumors have potential as biomarkers for diagnosis and prognosis, and also offer potential therapeutic targets.^273^ However, it’s worth noting that there is considerable heterogeneity in glycosylation patterns among different types of CNS tumors. This variability reflects the diverse origins and characteristics of these tumors and has implications for treatment and prognosis. The study of glycosylation in CNS tumors is complex due to the intricate nature of the brain and its environment.

##### Neuroendocrine neoplasms

Research on glycosylation in neuroendocrine neoplasms (NENs) highlights its critical role in tumor behavior and patient outcomes. NENs display altered glycosylation patterns, including changes in *N*-linked and *O*-linked glycosylation, which influence tumor proliferation, differentiation, and metastasis.^274^ These glycosylation changes also present potential as diagnostic and prognostic biomarkers, and offer novel therapeutic targets, particularly in disrupting tumor growth and spread. Additionally, glycosylation in NENs intersects with hormonal pathways and regulating the immune response, impacting the efficacy of hormonal and immunotherapies.^275^ The heterogeneity in glycosylation patterns among different NENs further underscores its significance in the pathogenesis and treatment of these tumors.

#### Sarcoma

Investigation of the roles of glycosylation in sarcoma, including osteosarcoma, is an emerging field that has already provided key insights. Sarcoma, like other cancer types, often displays altered glycosylation patterns. These changes can significantly impact tumor growth, invasion, and metastasis. Specifically, modifications in the glycosylation of cell adhesion molecules may enhance sarcoma cells’ ability to metastasize by altering their interaction with the surrounding environment.^265,276^ Glycosylation also affects sarcoma cells’ interactions within the tumor microenvironment, influencing growth and therapeutic response.^277,278^ In osteosarcoma, glycosylation is believed to contribute to disease pathogenesis and progression, but the detailed mechanisms and effects are less clear compared to other cancers. The field is still evolving, and further research may provide novel diagnostic and treatment approaches for this disease.

### Glycosylation in autoimmune diseases

Autoimmune diseases are caused by the immune response to autoantibodies, which resulting in damage to tissues.^279^ Immunoglobulin (Ig), the most abundant glycoprotein in serum, plays a crucial role in humoral immunity, and glycosylation can regulate the function of Ig-mediated cellular and humoral responses. These glycosylation changes can modify immunoglobulin G (IgG)’s binding affinity to Fcγ fragment receptors (FcγR) or C1q complement, thus influencing IgG’s role in regulating immune cell activities towards pro- or anti-inflammatory responses.^280^ Specifically, the IgG1 Fc segment at Asn-297, composed of GlcNAc and mannose, can undergo further glycan modifications. Such alterations in the Fc segment’s glycosylation can impact its affinity for FcγR, with reduced sialylation, for instance, enhancing pro-inflammatory FcγR activation.^281–283^

#### Rheumatoid arthritis

Rheumatoid arthritis (RA), a long-term autoimmune disorder that affects the joints, shows significant glycosylation alterations in IgG molecules, particularly in the Fc region, marked by reduced galactose and sialic acid. These changes enhance pro-inflammatory responses and are linked to RA’s severity.^283^ Additionally, these glycosylation variations, influenced by genetic and environmental factors, open avenues for personalized medicine in RA management. Compared with healthy controls, RA patients showed specific glycosylation patterns, such as lower galactosylation, lower sialylation, and higher IgG fucosylation.^284,285^ The first report on the abnormal *N*-glycosylation of serum IgG in patients with RA was published in 1985.^286^

For diagnosing RA, rheumatoid factor (RF) and anti-cyclic citrullinated polypeptide antibodies are the most reliable autoantibodies. Despite being clinically manageable, RA lacks a cure, underscoring the need to investigate its pathogenesis. Low expression of *N*-glycosylated sialylation is a pathogenetic feature of immunoglobulin to in RA patients.^282,287^ A study comparing IgG glycosylation in RA patients and healthy individuals revealed that IgG RF with decreased sialylation correlates with heightened RF activity. Additionally, elevated serum C-reactive protein levels intensify arthritic symptoms.^288^ In contrast, anti-citrullinated protein antibodies exhibit abnormally low sialylated Fc glycans, promoting osteoclastogenesis and susceptibility to inflammatory bone loss.^289,290^ However, studies found no significant link between IgA glycosylation and RA activity.^291^

The synovium, a specialized mesenchymal tissue in the joint cavity, is maintained by synovial fibroblasts (SFs), which preserve a homeostatic environment in healthy joints but become pro-inflammatory in RA.^292^ This transformation is linked to a decrease in the cytokine TNF-dependent glycosyltransferase ST6Gal-I and α2-6 sialylation, altering glycosylation and turning the synovial membrane pro-inflammatory.^293^ The shift may be due to healthy SFs having high levels of core fucosylation or terminal sialylation, regulated by TNF. This results in reduced sialylation of CD90+ SFs in the sub synovial layer, contributing to the pathological changes observed in RA.^294^

#### Systemic lupus erythematosus

Systemic lupus erythematosus (SLE) is classified as an autoimmune disease involving innate and adaptive immunity, characterized by symptoms like rashes, nephritis, and arthritis, largely due to excessive autoantibodies and immune complexes, notably anti-double-stranded DNA (anti-dsDNA) antibodies.^295,296^ Despite its high mortality and relapse rates, specific preventive or therapeutic measures for SLE are lacking. Research into altered glycosylation in SLE seeks to understand its mechanisms. A study utilizing quantitative mass spectrometry for glycan chain analysis, along with cluster analysis and machine learning, assessed the link between glycosylation of anti-dsDNA IgG and SLE activity. A significant positive correlation was observed between the level of fucosylation in anti-dsDNA IgG1 and SLE disease activity.^297^

Defects in *N*-glycosylation is associated with immune system dysregulation in SLE patients. The enzyme alpha-Mannosidase II, crucial for complex *N*-glycosylation through mannose residue removal, plays a key role. Mice deficient in alpha-Mannosidase II exhibit *N*-glycosylation anomalies, leading to an autoimmune disease resembling human SLE.^298^ Additionally, a study on lupus nephritis revealed that renal tissues from lupus nephritis patients are rich in mannosylation, a feature absent in other renal diseases. This is thought to result from inadequate complex *N*-glycosylation combined with mature *O*-glycosylation.^299^ Additional research suggests that complex *N*-glycosylation plays a protective role against autoimmune diseases like SLE by preventing endogenous non-autoimmune signals and reducing sterile inflammatory reactions.^300^

#### Inflammatory bowel disease

Inflammatory bowel disease (IBD) is a group of chronic inflammatory bowel diseases characterized by a chronic and prolonged course, mediated by a combination of genetic, immune, and environmental factors, including ulcerative colitis (UC) and Crohn’s disease (CD).^301^ The development of IBD is associated with an elevated expression of truncated *O*-glycans and changes in the expression of terminal glycan structures.^302^ A study compared plasma *N*-glycosylation levels between IBD patients and healthy individuals. Plasma specimens from patients with IBD were found to be characterized by more macroglycans, reduced relative abundance of heterodimeric and high-mannose structures, lower fucosylation, lower galactosylation, and higher levels of sialic acidification.^303^ Elevated IgG agalactosylation (G0), decreased IgG monogalactosylation (G1) and digalactosylation (G2) were found in IBD patients.^304,305^ In a retrospective analysis of 3441 plasma samples from patients with IBD, it was found that CD patients showed an increase in core fucosylation of IgG1 and IgG2/3, while UC patients exhibited a decrease in core fucosylation of IgG2/3.^305^ The severity of colitis in chemical and T cell-transfer-induced IBD mouse models was found to be less pronounced in Fut8 knockout mice. This finding provides further evidence supporting the involvement of Ig fucosylation in the development of IBD.^306^ Sialylation is another possible Ig glycosylation pattern involved in IBD development. Decreased IgG sialylation levels were observed in both CD patients and UC patients.^305^

In addition, there was a significant correlation between antibody glycosylation and disease activity in patients with IBD. For example, increased IgG2/3 agalactosylation, decreased IgG2/3 digalactosylation, or sialylation was associated with disease worsening in CD patients. Furthermore, elevated IgG1 agalactosylation or increased agalactosylated and fucosylated IgG to digalactosylation and fucosylated IgG ratio (G0F/G2F) associated with more extensive in CD patients.^305,307^ CD patients who received infliximab treatment exhibited a significant reduction in the agalactosylation of IgG.^308^

Despite advances in understanding IBD, the role of T cells’ glycosylation-modified structures remains largely unexplored.^309^ In IBD, memory CD4+ T cells produce colitis-associated glycoprotein, featuring immature *O*-glycosylation. CAG aids in forming CD4+ T cell immune synapses, thus promoting memory CD4+ T cell expansion in the inflamed gut and intensifying intestinal inflammation.^310^ Additionally, a study in IBD mice found increased core fucosylation on T-cells, essential for signaling, inflammatory factor production, and intestinal inflammation induction.^306^ Disruption of glycosylation promotes inflammation by perturbing intestinal barrier function, gut microbiota, glycan-lectin interactions, as well as mucosal immunity in IBD.^302^ These findings suggest that both *N*- and *O*-glycosylation are crucial in IBD’s pathogenesis, highlighting potential therapeutic targets in glycosylation inhibition.

#### Autoimmune liver disease

Autoimmune hepatitis (AIH) is a parenchymal inflammation of the liver mediated by an autoimmune response against hepatocytes, including primary biliary cholangitis (PBC) and primary sclerosing cholangitis (PSC).^311^ AIH is characterized by serum autoantibody positivity, hyperimmunoglobulin G and/or gamma-globulinemia, and the presence of hepatic histology of interfacial hepatitis, with a variety of clinical manifestations.^312,313^ A site-specific glycomic analysis of Ig in patients with autoimmune cholestatic liver diseases demonstrated elevated levels of IgG1 agalactosylation and reduced levels of IgG1 galactosylation in both PBC and PSC patients.^314^ PSC patients exhibited higher levels of IgG2 fucosylation compared to PBC patients. Conversely, decreased glycosylation of IgG3/4 was observed in PBC patients. In addition, elevated IgA 1/2 bisecting glycoforms, declined IgA diantennary glycoforms, and increased IgA 1/2 asialylation levels were observed in PBC patients.^314^ The presence of IgG2 agalactosylation showed a positive correlation with various stages of PBC,^314^ indicating the potential significance of Ig glycoforms as biomarkers and for monitoring disease progression in autoimmune cholestatic liver diseases.

Another study revealed the regulatory role of glycosylation in the pathogenesis of autoimmune hepatitis by establishing an *O*-GlcNAc glycosylation-deficient rat model.

The severity of AIH is linked to the activity of CD4+ and CD8+ T lymphocytes.^315^ While the exact role of glycosylation in AIH is unclear, recent studies have shed light on its significance. Research using an *O*-GlcNAc glycosylation-deficient rat model revealed that a lack of this glycosylation intensifies liver damage and prolongs recovery in AIH. This glycosylation deficit increases CD4+ T cell infiltration and impacts Treg differentiation, thus disrupting the Notch signaling in CD4+ T cells and accelerating AIH progression.^316^ Further, *O*-glycosylation inhibitors have been shown to worsen AIH, potentially due to altered cytokine levels and T-cell proliferation.^317^

#### Myasthenia gravis

Myasthenia gravis (MG) is an autoimmune disease that affects neuromuscular transmission. The most common subtype of MG is the production of autoantibodies directed against the nicotinic acetylcholine receptor (AChR) and is controlled primarily by the IgG1 and IgG3 subclasses.^318,319^ In addition to this, autoantibodies against muscle-specific tyrosine kinase (MuSK) are also present in many patients.^320^ Unlike AChR MG, MuSK MG isoforms are most often associated with IgG4 subclass autoantibodies.^321,322^ The level of Fab glycosylation of IgG4 antibodies was significantly elevated in MuSK MG, but this change was not found in the IgG subclass of AChR MG. One study explains that it may be related to the fact that the MuSK antigen is positively charged and the AChR antigen has a negative charge.^323–325^

MG patients have an increased frequency of *N*-linked glycosylation of IgG V regions (IgG-V^N-Glyc^) which may be associated with biased use of V gene fragments and somatic hypermutation. However, it has been experimentally confirmed that the binding of MG autoantigen-specific monoclonal antibodies (mAbs) to autoantigens is not altered by glycosylation.^326^ One study evaluated for the first time the changes in IgG Fc *N*-glycosylation in MG, observing low levels of IgG2 galactosylation.^327^

#### Nephropathy

IgG glycosylation plays an important role in the occurrence of kidney injury. Galactosylation, sialylation, and level of bisecting GlcNAc of the IgG glycans significantly correlate with renal function.^328^ One of the causative mechanisms of IgA nephropathy may be abnormal *O*-glycosylation of IgA1. The IgA1 *O*-glycosylation chain is truncated, resulting in increased terminal GalNAc and insufficient galactosylation.^329^ This overproduction and increased systemic presence of low *O*-glycosylated galactose-deficient IgA1 is recognized by anti-glycan autoantibodies, which form macromolecular IgA1 immune complexes that deposit in the glomerular mesangium, leading to renal inflammation and scarring.^330^

### Glycosylation in other diseases

In addition to the above alterations in glycosylation in tumors and autoimmune diseases, this PTM is increasingly being studied in metabolic and cardiovascular diseases. For example, it has been found that patients with type 1 diabetes mellitus have a relatively low abundance of simple dual antennae *N*-glycan (NA2), while complex multibranched, galactosylated, and sialylated modifications are characterized by a higher relative abundance of *N*-glycans.^331^ In chronic thromboembolic pulmonary hypertension, the distribution of IgG galactosylation is closely associated with N-terminal pro-B-type natriuretic peptide, demonstrating the pro-inflammatory properties of IgG *N*-glycosylation.^332^ In summary, glycosylation is involved in the occurrence and development of a variety of diseases. Further exploration of the value of glycosylation in disease diagnosis and prognosis is expected to provide a new choice for treatment strategy.^333^

#### Cardiovascular diseases

Glycosylation plays a role in transcriptional regulation, signal transduction, and energy metabolism, and is closely related to the development of cardiovascular disease (CVD). Among them, the role of *O*-GlcNAcylation in CVD is very important, but the mechanism is still not completely clear.^334^ Several studies have suggested a potential role for *O*-GlcNAcylation in cardiomyopathy through regulation of mitochondrial energy, autophagy, or gene expression.^335,336^ Decreased activity and expression levels of peroxisome proliferator-activated receptor-γ coactivator-1α (PGC-1α) when modified by *O*-GlcNAcylation and down-regulation of its expression in the myocardium may be involved in the formation of cardiac hypertrophy.^337^ Chen et al. also confirmed that the increase of *O*-GlcNAcylation in cardiomyocytes is related to myocardial hypertrophy, and hyperglycemia and hyperlipidemia can lead to the enhancement of protein *O*-GlcNAcylation, thus suggesting the importance of dietary control in the treatment of myocardial hypertrophy.^338^ In addition to cardiomyopathy, *O*-GlcNAcylation has also been shown to be associated with coronary microvascular disease.^339^ Reduced degradation of P53 protein modified by *O*-GlcNAcylation induces coronary microvascular disease and impairment of myocardial contractility.^340^ *O*-GlcNAcylation-modified synaptosome-associated protein 29 interferes with autophagic responses and impairs myocardial diastolic function.^341^

Changes in protein glycosylation could affect the interactions between blood cells and basement membranes or soluble plasma proteins, modulating blood viscosity, promoting local cell adhesion and inducing pro-inflammatory responses, leading to plasma cell extravasation, which is an important determinant of atherosclerosis and myocardial infarction.^342–344^ For example, the shear stress of sialidase affects cellular desialylation, leading to cell membrane stiffness and increased susceptibility to lysis and inflammation.^345^

Glycosylation of IgG is also associated with the pathophysiology of myocardial infarction. Some analyses have shown decreased serum galactosylation and increased levels of sialylation, fucosylation, and N-acetylglucosamine in patients with myocardial infarction.^346^ Three IgG *N*-glycosylation types associated with CVD were identified in a prospective study, with gender-specific associations.^347^ In conclusion, *N*-glycosylation of IgG may affect the risk of CVD, which may be an additional therapeutic target.

The key to clinical intervention in CVDs, such as myocardial infarction and ischemic stroke, is the application of thrombolytic enzymes in the acute phase to dissolve clots. Although drugs such as alteplase and urokinase are widely used today, they still suffer from insufficient clot dissolution rate and high risk of side effects.^348^ Glycosylation engineering could play a role in improving thrombolytic enzyme performance and resolving glycan covalent linkages.^349^ For example, tenecteplase, the most successful glycosylated engineered variant of alteplase, carries a total of six point-mutations in three different regions of the original base: Thr103Asn, Asn117Gln, and tetra-alanine substitution Lys296Ala+His297Ala+Arg298Ala+Arg299Ala.^350^ These glycosylation sites lead to a longer half-life of tenecteplase and significantly improved fibrin selectivity.^351,352^

#### Metabolic diseases

The incidence of metabolic diseases is increasing as a result of rapid economic development, population ageing and dietary adjustments. Metabolic diseases have a long course, affect the functioning of systemic systems, which means the prevention and control of these diseases will be of great benefit. Glycosylation, as one of the common types of PTMs, plays an important role in the development of metabolic diseases.

The EPIC-Potsdam study established a weighted score consisting of five IgG *N*-glycosylation characteristic glycans of type 2 diabetes and validated the association between the model and the risk of developing type 2 diabetes.^347^ IgG *N*-glycosylation is strongly associated with the incidence of diabetes. *O*-GlcNAcylation regulates pancreatic β-cell survival and function and affects insulin secretion. Decreased total insulin content and impaired glucose tolerance were found in transgenic mice overexpressing OGA in pancreatic islet β-cells in young month-old mice. With increasing months of age, *O*-GlcNAcylation in pancreatic islets increased and β-cell function was gradually restored.^353^ This suggests an important effect of *O*-GlcNAcylation on pancreatic islet function. *O*-GlcNAcylation is also a nutrient-sensing response, and studies have shown that induced removal of OGT decreases *O*-GlcNAcylation levels of lipid droplet-associated perilipin 1, leading to enhanced visceral lipolysis.^354^ Targeting OGT emerges as a potential target for the treatment of obesity.

Glycosylation also plays a role in lipid metabolism. It has been found that apolipoproteins, one of the key HDL-associated proteins, contain seven mucin-type *O*-glycosylation sites that correlate with HDL functional capacity and are not affected by short-term diet.^355^ The GALNT2 gene has been identified as a plasma lipid-related locus that encodes GalNAc-T2, which catalyzes GalNAc linkage in *O*-glycosylation and is a regulator of high-density lipoprotein cholesterol (HDL-C) metabolism.^356^ GALNT2 deficiency decreases the levels of HDL-C and phospholipid transfer protein (PLTP).^357^ Perhaps this suggests that GALNT2 affects HDL-C levels by regulating PLTP, which provides new therapeutic directions for dyslipidemia.

#### Neurodegenerative diseases

Normal brain function depends on protein homeostasis and high levels of glucose. The nutrient-sensitive *O*-GlcNAcylation pathway senses changes in glucose levels and utilizes glucose to convert and synthesize it into UDP-GlcNAc, which is added to proteins and completes PTM of proteins.^358^ This has been linked to the deposition of protein aggregates in neurodegenerative diseases such as Alzheimer’s disease(AD), Parkinson’s disease(PD), and Huntington’s diseases.^359^

One of the characteristic changes in the brains of AD patients is that the microtubule-associated protein tau is abnormally hyperphosphorylated and aggregates into neurofibrillary tangles, and it has been found that this may be based on reduced levels of *O*-GlcNAcylation.^360^ A low glucose uptake-induced decrease in *O*-GlcNAcylation, which negatively regulates tau phosphorylation in a site-specific manner in vitro and in vivo, occurs in a mouse model mimicking AD, leading to excessively elevated levels of its phosphorylation.^361^ The OGA inhibitor Thiamet-G was also shown to block tau protein hyperphosphorylation in vivo.^362^ Another characteristic change in the brain of AD patients is the deposition of β-amyloid (Aβ) as neuritic plaques, and it was found that *O*-GlcNAcylation enzyme inhibitors could also inhibit this process. Kim et al. applied the *O*-GlcNAcylation enzyme inhibitor, NButGT, to AD mice, and analyzed that NButGT acted on the S708 residue of nicotinic acid proteins to reduce the activity of γ-secretase and decrease the production of Aβ.^363^ The above results suggest that *O*-GlcNAcylation may be a potential target for the treatment of AD.

However, data from different models of neurodegenerative diseases suggest that increased levels of *O*-GlcNAcylation can also have a negative effect on disease. For example, in a model of Caenorhabditis elegans models, the absence of OGT enzyme activity associated with increased *O*-GlcNAc levels attenuates proteotoxicity.^364^ The characteristic manifestation of PD is Lewy bodies, the formation of cytoplasmic perinuclear inclusion bodies in neurons, which are predominantly α-synuclein.^365^ *O*-GlcNAcylation may affect the degradation of α-synuclein. By inhibiting OGA enzyme activity associated with reduced *O*-GlcNAc levels, total α-synuclein levels are increased.^366^ In conclusion, *O*-GlcNAcylation has been associated with the progression of a variety of neurodegenerative diseases, and it is crucial to study its regulation in depth and to grasp the pathophysiological process of diseases caused by *O*-GlcNAcylation dysregulation.

## Diagnostic and prognostic value of glycosylation in diseases

### Potential diagnostic value of glycosylation as a biomarker

As mentioned above, abnormalities in glycosylation are crucial in several human diseases. Accumulating studies indicated the value of glycosylation in disease diagnosis and prognosis.^367^ A variety of glycoproteins have been used as tumor markers in clinical diagnoses and predictor activities, such as alpha-fetoprotein in liver cancer, prostate-specific antigen (PSA) in prostate cancer, or glycol-residue-related markers, such as CA19-9 in pancreatic cancer.^241,368^

Combining serum IgG galactosylation with quantitative changes in CA125 may provide a more effective differential diagnosis for ovarian cancer, with the diagnostic specificity improved from 65.2% (CA125 test alone) to 84.6%.^369^ The large-sample-based analysis further confirmed that the distribution of IgG galactosylation could serve as a non-invasive pan-cancer biomarker for early cancer detection and cancer screening.^370^ The diagnostic and prognostic value of glycosyltransferase-related genes were evaluated in several tumors, which may also provide good biomarkers for individualized medicine.^244,371^

In addition, several studies have indicated the prognostic value and clinical relevance of ST6Gal-I. ST6Gal-I was demonstrated to enhance HIF-1α signaling, thereby protecting ovarian and pancreatic tumor cells against hypoxic stress.^372^ The glycosyltransferase ST6Gal-I is upregulated in ovarian and pancreatic cancers and expressed at high levels in metastatic tumors.^373^ In addition, the glycosylation patterns of glycoproteins associated with circulating extracellular vesicles, as well as mucins, present potential targets for the development of detection methods. Detection of glycosylation cancer-associated mucin 1, mucin 16 and CD63 may contribute to the accurate and effective differentiation of primary breast cancer.^374^ Inadequate glycosylation of MUC1 leads to exposure to novel epitopes that can be used as specific targets for treatment and diagnosis.^375^ The subcellular localization of tumor-associated MUC1 is significantly different from that of normal cells. Alirezapour et al. synthesized 64Cu-DOTA-PR81, a tracer by combining 64Cu labeling with PR81(a high-affinity mAb to MUC1), and showed high sensitivity and specificity in tumor diagnosis.^375,376^

In addition to the detection of markers in serum, the specific glycoproteins contained in urine and saliva, which are non-invasive specimens, would simplify the screening and monitoring of pathologies.^377,378^ The combination of glycosylation detection and current diagnostic tools can better manage diseases and promote accurate diagnosis and treatment of diseases and efficacy monitoring.

### Potential prognostic value of glycosylation

A variety of glycosylation-modifying proteins have been used for cancer-specific diagnosis, meanwhile, several studies have explored the prognostic value of GTs expression levels in cancer. Recently, a prognostic signature in HCC based on six GTs was established, which showed a good performance in predicting OS.^379^ Seventeen glycosyltransferases, which play a role in the initial stages of *N*- or *O*-glycosylation, as well as glycolipid biosynthesis and sialylation, were unequivocally identified as being associated with a poor prognosis in malignancy. Among them, ALG3, GALNT2, B4GALNT1, POFUT1, B4GALT5, B3GNT5 and ST3GAL2 as the most consistently malignancy-associated enzymes, compared to the published experimental data points.^380^

In addition to glycosyltransferases, characteristic glycosylation of IgG can serve as a prognostic biomarker. Theodoratou et al. demonstrated that IgG with reduced galactosylation, reduced levels of sialylation, and increased bifurcated GlcNAc was strongly correlated with all-cause and colorectal cancer mortality, with a good predictive effect.^381^ FUTs are also considered as an essential biomarker for the detection and prediction of several types of cancer, because altered protein fucosylation is a characteristic of cancer. Overexpression of FUT3 is linked to reduced overall survival in TNBC patients.^382^ FUT8 was significantly associated with poor survival in non-small cell lung cancer.^383^

In addition, the expression of glycosylation genes may be associated with cancer prognosis.^384^ Zhao et al. identified three subtypes of glycosylation-regulated genes that are highly compatible with the immunophenotypes of HCC and further constructed glycosylation-related model scores.^385^ Another study, also for HCC, used bioinformatics analysis to construct a five-gene signature that predicted the prognosis of patients with HCC and delineated risk stratification.^386^ In lung adenocarcinoma, a risk model based on differentially expressed GTs-related genes was conducted, which could predict the survival rate of patients.^387^ Both the use of GTs as effective prognostic markers for tumors and the construction of a genetic prognostic model for glycosylation exemplify the potential of glycosylation in assessing cancer prognosis. It is important to explore the potential of glycosylated proteins as biomarkers for human diseases (Table 1).

**Table 1:** Diagnostic and prognostic biomarkers related to glycosylation

Biomarker	Cancer type	Glycosylation	References
AFP	liver cancer	Core fucosylated biantennary or hybrid glycans or bisecting glycans↑	^486^
CA125(MUC16)	ovarian cancer	Core-fucosylated biantennary monosialylated glycans↑	^487,488^
CA15-3(MUC1)	breast cancer, ovarian cancer	Truncated *O*-glycans↑	^489–491^
CA19-9	gastric cancer, pancreatic cancer, colorectal cancer, ovarian cancer	Sialylation↑	^492–495^
CEA	breast cancer, colorectal cancer, pancreatic cancer	Highly heterogeneous in sialylation and fucosylation	^496–499^
CP	liver cancer, pancreatic cancer	Fucosylation↑	^500,501^
hAGP	pancreatic cancer	Core-fucosylation↑	^502^
Hp	liver cancer, ovarian cancer	Fucosylation↑	^252,503,504^
IgG	liver cancer, gastric cancer, lung cancer, colorectal cancer	Galactosylation ↓ , sialic acids and more complex highly branched *N*-glycan ↑ ; α-2,6-sialylated fucosylated *N*-glycans↑	^505–508^
PSA	prostatic cancer	Core 2 *O*-sLe(x)↑, Neu5Ac α2-6 sialylated glycans ↑ , galactosylated glycans ↑ , N-acetylgalactosaminylated glycans↑	^449,509^
RNase 1	pancreatic cancer	Core-fucosylation↑	^510^
PON1	liver cancer, head and neck cancer	Fucosylation↑	^511,512^
sLeX	lung cancer, breast cancer	Sialylation ↑ , Core-fucosylation↓	^513–515^
STn	gastric cancer, ovarian cancer	Truncated *O*-glycans↑	^491,516^
A1AT	liver cancer, lung cancer	Core-fucosylation↓	^517,518^

*AFP* alpha-fetoprotein, *CA125* carbohydrate antigen 125, *MUC16* mucin 16, *CA15-3* carbohydrate antigen 153, *CA19-9* carbohydrate antigen19-9, *CEA* carcinoembryonic antigen, *PSA* prostate-specific antigen, *RNase 1* ribonuclease 1, *CP* ceruloplasmin, *hAGP* human α1-acid-glycoprotein, *HP* haptoglobin, *IgG* immunoglobulin G, *PON1* serum paraoxonase 1, *sLeX* sialyl-Lewisx, *STn* sialyl-Tn, *A1AT* α1-Antitrypsin

## Targeting glycosylation for the treatment of diseases

Glycan-based therapies are approaches with great potential in the treatment of cancer, including protein glycosylation inhibitors, small molecule inhibitors of glycosyltransferases, glycosylation-modified mAbs, glycosylation-mediated targeted drug delivery, and vaccine design (Table 2). The choice between glycosylation-related targeted treatments and other targeted treatments depends on the specific molecular characteristics of the tumor, the general health status of patients, and the potential benefits and risks.

**Table 2:** Strategies targeting glycosylation in clinical trials

Approach	Target	Drug candidate	Cancers	Phase	Identifier
Inhibitors of glycosylation	Galectin-1	OTX008	solid tumors	I	NCT01724320
	Galectin-3	GB1211, GCS-100	metastatic melanoma, head and neck squamous cell carcinoma, non-small cell lung cancer, diffuse large B-cell lymphoma, chronic lymphocytic leukemia	I/II	NCT05913388, NCT05240131 NCT00776802, NCT00514696
	Galectin-1 and -3	GR-MD-02, GM-CT-01	melanoma, non-small cell lung cancer, squamous cell carcinoma of the head and neck, colorectal cancer, cancer of the bile duct, gallbladder cancer	I/II	NCT02575404, NCT00110721 NCT00054977, NCT01723813 NCT02117362, NCT00388700 NCT00386516
	Selectins	GMl-1271	acute myeloid leukemia, multiple myeloma	I/II/III	NCT03616470, NCT02811822 NCT02306291
	Fucosylation	SGN-2FF	non-small cell lung cancer, renal cell carcinoma, breast neoplasms, urinary bladder neoplasm	I	NCT02952989
Glycan-based antibodies	Gangliosides (e.g., GD2, GD3, GM2, GM3 and fucosyl- GM1)	hu3F8, ch14.18, hu14.18- IL2, PF-06688992, KW- 2871, BMS-986012	neuroblastoma, lung cancer, osteosarcoma, melanoma, brain and central nervous system tumors, ovarian cancer, retinoblastoma, sarcoma, small intestine cancer, melanomas, other solid tumors	I/II/III	NCT00089258, NCT00002458, NCT01183897, NCT01704716 NCT00026312, NCT03363373 NCT01757626, NCT04560166 NCT02484443, NCT02502786 NCT00590824, NCT00450827 NCT00445965, NCT03860207 NCT04750239, NCT03159117 NCT00199342, NCT00679289 NCT02815592, NCT02247349 NCT04702880
	Globo H	OBI-888, OBI-999	solid tumors	I/II	NCT03573544, NCT04084366
	Truncated O-glycan (e.g., Tn, and STn)	PankoMab-GEX™	ovarian epithelial cancer, fallopian tube cancer, primary peritoneal cancer, breast cancer	I/II/III	NCT01222624, NCT01899599 NCT03360734
	Lewis antigens (e.g., LeA, and LeY)	MVT-5873 hu3S193	pancreatic cancer, ovarian cancer, small cell lung cancer	I/II	NCT02672917, NCT03801915 NCT00084799, NCT00617773 NCT00051571
	MUC-1	TAB004, SAR566658, MUC1 + CD3 BsAb	breast cancer	I/II	NCT04137900, NCT01156870 NCT02984683, NCT03524261
Glycan-based vaccines	Glycolipids (e.g., GD2, GD3, GM2, GM3, and α-galactose containing)	bivalent vaccine, GM2-KLH vaccine, BEC2 Vaccine, BCG vaccine	sarcoma, neuroblastoma, small cell lung cancer, breast cancer, fallopian tubes, ovarian cancer, peritoneal cancer, metastatic melanoma	I/II/III	NCT01141491, NCT00911560 NCT01349647, NCT00003357 NCT01248273, NCT00668512 NCT00037713, NCT00006352
	Globo H	OBI-822	breast cancer	II/III	NCT03562637
	Truncated O-glycan (e.g., T, Tn and STn)	MAG-Tn3 + AS15, THERATOPE STn-KLH vaccine	ovarian cancer, peritoneal cancer, breast cancer, prostate cancer	I/III	NCT01248273, NCT00003638 NCT00003819, NCT02364492
	Lewis antigens	sialyl Lewis A-KLH + QS21	breast cancer	Not Applicable	NCT00470574
	Polysialic acid	Polysialic acid-KLH + QS21	small cell lung cancer	II	NCT00004249
	MUC-1	YB-01	breast cancer, other solid tumors	I/II	NCT03014076, NCT03384316 NCT05986981
	Polyvalent vaccines	(Bivalent vaccine+ OPT-821), (Trivalent vaccine- KLH conjugated + OPT-821), (Globo-H-GM2-STn-TF-Tn-KLH-QS21), (GD2L, GD3L, Globo H, fucosyl- GM1, and N- propionylated polysialic acid -KLH + OPT-821)	neuroblastoma, sarcoma, ovarian cancer, breast cancer, small cell lung cancer	I/II	NCT00911560, NCT01141491 NCT01248273, NCT01349647
CAR-T cells against aberrant glycosylation	Gangliosides (e.g., GD2)	GD2Bi-aATC, 4SCAR-GD2, 1RG-CART, GD2-CART01, GINAKIT	neuroblastoma, sarcoma	I/II	NCT02173093, NCT02765243, NCT03356782, NCT02992210 NCT02761915, NCT02765243 NCT03373097, NCT03294954
	Lewis antigen (e.g., LeY)	LeY-CAR-T	lung cancer, fallopian tube cancer, other solid tumors	I/II	NCT03198052, NCT03851146
	MUC-1/MUC1-Tn	MUC-1 CART huMNC2-CAR44	intrahepatic cholangiocarcinoma, breast cancer, lung cancer, ovarian cancer, fallopian tube cancer, multiple myeloma, pancreatic ductal adenocarcinoma	I/II	NCT03633773, NCT03179007 NCT04020575, NCT05812326 NCT03525782, NCT04025216

*MUC-1* mucin 1, *CAR* chimeric antigen receptor, *STn* sialyl-Tn

### Inhibitors of protein glycosylation

Inhibitors of the glycosylation mechanism have been proved to be efficacy in cancer treatment. Among them, most are small-molecule proteins that are readily taken up by cells, thus providing an opportunity for researchers to design drugs.^388^ A variety of protein glycosylation inhibitors are currently under investigation or have been put into clinical use as effective strategies to block cancer progression. Per-*O*-acetylated GlcNAcbeta1,3Galbeta-O-naphthalenemethanol inhibits sLeX in tumor cells and inhibits hematogenous dissemination of mouse lung cancer cells.^389^

Increased aerobic glycolysis characterizes cancer cells, and 2DG, as a glycolysis inhibitor, became an effective strategy to cause cancer cell death, yet not through direct inhibition of glycolytic processes. 2DG treatment of lymphocytic choriomeningitis virus resulted in a dramatic reduction of pathogenic particles, which was further demonstrated to be done by inhibiting the *N*-glycosylation of viral glycoproteins.^390^ 2DG impairs the *N*-glycosylation of the key oncogenic receptors Axl and Met in SCC15 cells, an oral squamous cell carcinoma cell line, decreasing cell viability and colony-forming ability.^391^ Thus, 2DG could be used with other drugs to synergistically treat the disease. In addition, 2DG in combination with metformin inhibits protein *N*-glycosylation and induces an unfolded protein response, which combined with adequate energy stress results in mitochondrial enlargement and improved anti-tumor immunity.^392^

Overexpression of mucin glycans is an important mechanism for cancer development, and inhibitors targeting mucin glycans would be very promising therapeutic directions.^393^ As an example, MUC1, which is currently the most well-characterized, expresses mainly *O*-glycosylation and *N*-glycosylation.^394^ Knockdown of *O*-glycosylation of MUC1 significantly restored chemosensitivity in wild-type WT breast cancer cells, whereas insertion of the *O*-glycosylation site increased drug resistance.^395^ MUC1-based antibodies, radiopharmaceuticals, vaccines and chimeric antigen receptor (CAR)-T cell therapy are continuously being developed. A mAb (TAB004) specifically targeting human MUC1 was developed, which binds to MUC1 to induce ER stress and anaerobic responses in PAAD cells and inhibit cell proliferation.^396^ Salouti et al. generated 99mTc-HYNIC-PR81 by radiolabeling PR81, an antibody specific for MUC1, with 99mTc, which improved the stability and immunoreactivity of PR81.^397^ DNA vaccine (pcDNA3.1-VNTR) controls tumor growth and extends survival time in mice by enhancing MUC1-induced CTL activity.^398^ Novel CAR-T cells targeting MUC1-Tn inhibit the growth of MUC1-Tn-positive cancer cells in leukemia and PAAD mouse models.^399^ In conclusion, targeted therapies based on inhibition of MUC1 glycosylation are promising, but further in-depth studies are needed to obtain more robust therapeutic responses.

### Small molecule inhibitors of enzymes

Glycosidases and GTs are key enzymes in the glycosylation process, and their expression levels regulate the function, properties, stability, and localization of glycosylated proteins. Several studies of small molecule inhibitors targeting these enzymes have been conducted, which provide novel strategies for cancer treatment.^400^ Shi’s team found that knocking down the MAN2A1 gene, which encodes the N-glycan maturation enzyme, resulted in the deletion of MAN2a1 in cancer cells, but increased the infiltration of tumor cytotoxic T-cells under anti-PD-L1 treatment.^401^ It further demonstrated the utility of *N*-glycosylation in combination therapy with immune checkpoint inhibitors. Pancreatic cancer ductal cells, referred to as Panc1 cells, express core 2 N-acetylglucosaminyltransferase-M. When Panc1 cells are subjected to heat shock, the non-muscle myosin IIA-C2GnT-M complex is increased, as is the polyubiquitination and proteasomal degradation of C2GnT-M, which induces Golgi disruption.^402^

Targeting the enzymes that play a role in angiogenesis and regulating the glycosylation process to achieve anti-angiogenesis, is also a new direction of glycosylation in cancer therapy. The α-glucosidase I inhibitor castanospermine converts high-mannose-type *N*-glycosylation to complex oligosaccharides, alters endothelial cell proliferation and attachment capacity, prevents angiogenesis, and inhibits tumor growth.^403^ The enzymatic activity of GnTs catalyzes the *N*-glycosylation pattern in proteins, thereby regulating the glycosylation status of key angiogenic factors such as VEGFR2 and Notch.^404^

### Glycosylation of mAbs

mAbs are biological agents that are widely used in various diseases and are highly regarded for their specificity, sensitivity and relatively low cross-reactivity. The structure and stability of glycosylation are crucial for the function of mAbs. Altering the Fc glycosylation structure enhances the antibody’s ability to attract innate immune responses.^405^ *N*-glycosylation is the most common form of glycosylation in mAb therapeutics.^406^ The CH2 domain of deglycosylated antibodies is less thermally stable, more susceptible to loss of higher structures in space and more susceptible to papain.^407^ Kayser’s team found that glycosylated mAbs are less stable and appear to improve solubility and stability, which may be important for long-term storage.^408^

The addition of 2-fluoroperacetylated fucose, an inhibitor of FUT, to cell cultures or transfection of EG2-hFc-producing cells with the prokaryotic GDP- 6-deoxy-D-lyxo-4-hexanone glucose reductase (RMD) gene resulted in a reduction in the level of fucosylation of the antibody glycan in CHO cells in both methods.^409^ This alteration enhances antibody-dependent cytotoxicity, Fcγ receptor binding, and affects antibody effector function. Glycosylation in mAbs and common antigens require further research to control these components in vivo and to selectively utilize glycosylation targeting. Moreover, deletion of the intramembrane protease signaling peptide peptidase-like 3, which resides within the Golgi, leads to hyperglycosylation of CD19, an alteration that directly inhibits effector function and suppresses antitumor cytotoxicity in CAR-T cells.^410^ Dysregulation of this PTM of glycosylation of the leukocyte differentiation antigen CD19 might be one of the mechanisms of antigen escape from CAR T cell therapy.

### Glycosylation-mediated targeted drug delivery

Glycosylated structures are an important part of the tumor cell membrane and selectively bind to the cell. Molecules such as mannose, galactose, and glucose are generally used to bind to the delivery system and are non-toxic, non-immunogenic, and have good histocompatibility.^411^ Therefore, targeted drug delivery systems based on glycosylated structures could control drug distribution, maximize therapeutic efficacy, and minimize unfavorable drug behavior.^412^ The lectin receptor is one of the targets of glycans, which is important in the immune system. For example, it establishes the first line of defense against microorganisms, recognizes self and non-self, and regulates inflammatory response processes.^413^ The lectin receptor and glucose transporter subtype I (GLUT1) are widely studied. Drug delivery systems and glycosidic ligands can form conjugates that selectively concentrate drugs at lesion sites. Lectin receptors exert their targeting properties through covalent binding to mannose and galactose.^414^ GLUT1-specific glycans bind to drug carriers to cross the blood-brain barrier for tumor-targeted drug delivery.^415^

Nanoparticles receiving galactosylation or mannosylation modifications loaded with the tumor-specific antigen SIINFEKL peptide (OVA) rapidly enhanced antigen uptake and dendritic cell maturation, and were able to promote the toxic effects of CD8+ tumor-infiltrating lymphocytes on lymphoma cells.^416^ A tobacco mosaic virus-based drug delivery system with a glycan structure (mannose or lactosylation modification) as the targeting ligand, loaded with the anticancer drug cisplatin, exhibited robust endocytosis and apoptosis in cell lines.^417^ The galactose-installed photo-crosslinked pH-sensitive degradable micelles system loaded with paclitaxel indexed significant tumor cell death with less damage to the liver and kidney.^418^ Targeting up-regulated glycan transporters in glioblastoma using glucose, mannose or galactose molecules as ligands conjugated to hydroxyl-terminated polyamidoamine dendrimers significantly improves targeting, specificity and aggregation kinetics of tumor macrophages in glioblastoma.^419^ Glycosylation-modified polymers are effective nanocarriers for loading anticancer drugs and have important cancer intervention implications. However glycosylated modified proteins may have multiple problems at the same time. For example, multiple recognition problems affect targeting efficiency. Conjugation strategies based on glycosylation may alter the characteristics of the delivery vehicle, such as surface charge or size. It may also produce off-target effects affecting other organs, etc., all of which require further in-depth studies.^420,421^

### Novel vaccines based on glycosylation

Vaccines prevent various diseases by inducing humoral and/or cellular immunity, with carbohydrates on cell surfaces playing a key role in both innate and adaptive immunity, making them attractive antigens for vaccine development. Covalent linking of carbohydrates to immunogenic proteins enhances their immunogenicity.^422^ A study integrated the prostate cancer cell antigen carbohydrate molecule globo H hexasaccharide with keyhole grouper blood blue protein, together with the immune adjuvant QS-21, to patients who have recurred from radiation or surgery.^423^ Synthesis of two- or three-component antitumor vaccines with glycopeptides of the tumor-associated mucin MUC1, T-cell epitope peptides, and Pam(3)CSK(4) lipopeptides also produced a strong immune response that induced the killing of tumor cells.^424^ Despite numerous glycosylation-based vaccines reaching clinical trials, they predominantly remain in vitro, and none have yet met clinical application criteria.^425^ Identifying optimal immune components and assembling effective vaccines to prevent or halt disease progression remains a significant challenge

### Glycoconjugate drugs

Despite the remarkable efficacy of most first-line anticancer drugs, the prevalence of “off-target” drug effects has led to the occurrence of serious adverse events such as alopecia, nausea, and vomiting, which have a significant influence on the patient’s quality of life and disease prognosis. To achieve better clinical efficacy of chemotherapeutic drugs, glycan conjugation methods of drugs have been figured out. Covalent conjugation of glycan structures to drugs improves drug compatibility, reduces toxicity, and minimizes adverse effects. In 1995, the first glycoconjugate drug, glufosfamide, was reported to be better tolerated, less toxic, and more efficacious, and entered clinical trials.^426^ Chloramphenicol with the addition of glycosylated structures such as *N*-alkoxyamine- and *N*-acylhydrazine- exhibits a broader tumor specificity, as well as greater potency.^427^ Mikuni et al. found that the in vivo potency of galactose-conjugated docetaxel in a mouse leukemia tumor model was superior to paclitaxel in terms of antitumor activity.^428^ The addition of polysaccharides to common drugs also enhances the drug’s ability for action. The antacid-alginate combination (under the trade name Gaviscon) is a drug that effectively inhibits gastroesophageal reflux disease by forming a gel that floats on top of the stomach contents. Conjugation with a glycan base greatly reduces the adhesion of Helicobacter pylori to the gastric epithelial cells and improves drug action.^429^ Glycoconjugated drugs often improve the water solubility and stability of the drug and may enable selective targeting of tumor cells.^430^

### How to design specificity for targeting glycosylated drugs

To deliver therapeutic drugs to cancer cells more effectively while minimizing toxicity and off-target effects of the drugs on normal cells, maximizing the eradication of cancer cells, and improving the specificity of targeted drugs, dual or multiple targeting strategies can be adopted. Systemic administration using dual-targeted heterodimers significantly improves DNA sensing, DC cross-presentation, and anti-tumor T-cell responses, and tumor cell-specific dual-targeting enables better tumor control.^431^ Dual/multi-targeted drugs are favorable to solve the problems of limited efficacy, poor safety and drug resistance that a single target has. Based on a full understanding of the structural biology of glycosylation, rational use of bioinformatics tools and proteomic information provides the possibility of designing highly effective and safe multi-targeted anticancer drugs.^432^

## Novel detection methods for glycosylation

Glycosylation has emerged as a variety of assays over the course of several years of research, which are briefly summarized below.

### Liquid chromatography and mass spectrometry

As mentioned earlier, biomarkers of glycosylation are of great value in tumor diagnosis and prognosis, and thus glycosylation analysis is a critical step. However, this is challenging because of the need to identify both glycosylation sites and glycan structures.^433^ Liquid chromatography (LC)is the most widely used technique in the separation of glycopeptides and released glycans.^434,435^ A study has revealed the high performance of three hydrophilic interaction liquid chromatography (HILIC) columns for the separation of human IgG glycopeptides, as well as the successful separation of the isomeric A2G1F1 glycopeptide of IgG and the structural potential of the isomeric glycopeptide.^436^ Hernandez-Hernandez’s group used HILIC with reversed-phase liquid chromatography to isolate the *O*-glycopeptide structure of bovine casein myopeptide.^437^ Although LC is the most commonly used separation technique for glycoproteomics analysis, it has its limitations. For example, the use of multiple solvents as mobile phases results in higher analytical costs, complicated operation and environmental pollution.

Mass spectrometry (MS) is a method of ionizing molecules in a sample and separating the ions in a magnetic or electric field. It has become a powerful tool in the analysis of saccharides and glycomacroproteomics.^438^ A high-throughput matrix-assisted laser desorption ionization mass spectrometry (MALDI-MS) method based on microarray technology for the successful detection of *N*-glycosylated peptides of IgG1 produced during in vitro cell culture.^439^ Some studies have used MS to detect glycosylation of serum glycoprotein transferrin and used *N*-glycosylation levels to determine subtypes of congenital disorders of glycosylation.^440^ Kotsias’ research group detected multiple *O*-glycosylation compositions of in vitro cancer cell lines using ultra-high resolution MALDI-FT-ICR MS, enabling semi-automated, high-throughput glycan identification and quantitative analysis.^441^ Some studies have used porous graphitized carbon chromatography coupled to tandem mass spectrometry to analyze the epitopes of *N*- and *O*-glycosylation in AML and to identify glycan profiles.^90^

MS ionization is inefficient and MS alone does not always provide complete structural and quantitative information, and needs to be combined with other techniques. For example, the separation step prior to MS, which mainly consists of LC and capillary electrophoresis methods, can more reliably identify and quantify glycans and glycopeptides. In a study using LC-MS/MS for glycosylation analysis, increased expression levels of high mannose and α2,6-linked glycosylated complex *N*-glycans were observed in colorectal cancer cell lines and tissues.^442^ Determination of glycosylation in glycan-conjugated vaccines using solid-phase extraction coupled with LC was performed, and tandem mass spectrometry detected 14 and 12 glycosylation sites in the CRM197 vaccine and tetanus toxin-based vaccine, respectively, and was achieved with high sensitivity and a wide linear dynamic range.^443^ Naumann’s group performed high-throughput glycosylation analysis of intact mAbs based on LC and MS in combination with capillary electrophoresis, and efficiently determined the five major glycosylation patterns of the tested antibodies.^444^ In addition to this, MS is capable of combining various techniques, such as fluorimetry, electrospray ionization, and isotope-specific labeling, for qualitative and quantitative studies of glycosylation and in-depth characterization.^445–447^

### Lectin-based techniques

In addition to the traditional approach of using LC to isolate and purify glycoproteins and then MS to identify glycans, a variety of lectin-based assays based on the interaction of glycans and glycan-binding proteins have been developed. For example, lectin microarrays, lectin chromatography and lectin immunosorbent assay are used to detect glycosylation patterns.^448,449^ The development of high-density lectin microarrays is critical for identifying lectins specific to disease-related protein glycosylation.^450,451^ Lectin microarrays enable rapid and simple analysis of glycosylation to determine the most appropriate lectin to enrich for glycoproteins.^452^ Although lectin microarrays provide data on lectin-glycoprotein interactions, they are unable to account for changes in glycosylation patterns of individual proteins or of glycoproteins as a whole and need to be combined with other techniques.^453^ Patwa et al. combined lectin microarrays with all-liquid-phase enrichment and pre-fractionation methods to discover differences in glycosylation between pancreatic cancer and normative human sera, particularly with respect to mannosylation and fucosylation.^454^ Early lectin detection techniques, such as tandem lectin affinity chromatography, did not meet the criteria for glycosylation analysis. Lectin microarrays and lectin immunosorbent assay, which analyze the structure of glycans in a comprehensive, high-throughput manner, are not only simple to perform, but also offer significant advantages in terms of sensitivity and reproducibility.^455^

### Electrophoresis

Conventional sodium dodecyl sulfate-polyacrylamide gel electrophoresis (SDS-PAGE) was unable to meet the criteria for separating glycosylation patterns, and more advanced techniques were developed. Previous studies have confirmed that two-dimensional gel electrophoresis. Which could analyze peptide mixtures, can be used for the analysis of glycosylated peptides.^456^ Polyacrylamide Gel Electrophoresis is a new technique introduced based on gel electrophoresis method for detecting the levels and dynamics of *O*-GlcNAc. Polyacrylamide Gel Electrophoresis can separate native *O*-GlcNAc proteins from their unmodified counterparts, does not require complex sample handling and advanced equipment beyond the electrophoresis apparatus, and has a high degree of specificity and affinity, making it particularly suitable for *O*-GlcNAc analysis.^457^ In addition, Liu et al. developed a new technique of supported molecular matrix electrophoresis to detect glycosylation patterns of Fut8+/+ and Fut8-/- mouse serum proteins, and further analyzed the *N*-glycosylation and *O*-glycosylation patterns in the isolated γ-bands by mass spectrometry. Supported molecular matrix electrophoresis is more economical in the amount of protein samples compared to SDS-PAGE, and the serum fractionation is simpler and faster, and the compatibility of glycoproteins with the separated bands.^458^

Capillary gel electrophoresis (CGE) has been used in the past in conjunction with LC and MS as a method for the structural separation of oligosaccharides because of its high performance in separation, resolution, and sample handling.^459^ Combined with laser-induced fluorescence decolorization, the CGE-based method has become a highly efficient analytical technique for the characterization of glycosylation patterns, with high performance in terms of low cost of separation, robustness, speed of analysis, and sensitivity.^460^ Multiplexed CGE using laser-induced fluorescence detection was able to monitor the structural properties of *N*-glycosylated glycans.^461^ It is of great value in the ability to identify small differences in glycosylation composition and structure.^462^

### Radiolabelling

While MS is able to identify the glycopeptide composition and glycan structure, it cannot obtain the accurate stereo-structure of glycans, and at the same time, it cannot distinguish the types of glycan residues with the same molecular weight.^463^ Schubert’s team proposed nuclear magnetic resonance (NMR) spectroscopy experiments to analyze glycosylation patterns.^464^ Structural changes in the *O*-glycosylation of calcitonin derivatives have been reported using NMR analysis, which revealed localized residue lengthening at the *O*-glycosylation site, thus disturbing the original α-helical structure of calcitonin.^465^ NMR allows the detection of glycan stereochemistry, composition and type of linkage bonds and quality control of the produced proteins, regardless of molecular size and without isotopic labeling.^466^ It has the disadvantage of not being able to identify modification sites in the protein sequence.

On the other hand, Raman imaging was conducted to monitor the glycosylation metabolism in brain and breast cancers. The researchers identified Raman markers based on the vibrational characteristics of glycogen, glycans, etc., and combined them with chemometrics to distinguish protein glycosylation patterns in different tissues.^467^ The structure of the secreted polymeric gel MUC5B was analyzed by Raman imaging and found to be rich in *O*-glycosylation.^468^ Raman imaging is a powerful visualization method that is sensitive and specific, does not require staining, and has the spatial and spectral resolution to provide important information to help analyze the multiple components present in a cell and map the distribution of glycans in tissues. The disadvantage of Raman imaging is the inability to quantify glycosylation levels.

### Molecular fluorescence

Fluorescence detection has been widely used in the identification of glycosylation structures because of its advantages of high sensitivity and direct observation. A series of fluorescent boron-dipyrromethene dyes can be incorporated and labeled with glycan groups during glycosylation structure synthesis, while relying on their chemical stability not to react with the glycan molecules. Meanwhile boron-dipyrromethene labeled glycans have high fluorescence efficiency in water.^469^ Zaro’s group has visualized the mucin-type *O*-glycosylation using in-gel fluorescence detection.^470^ However, there is also the problem that some fluorescent dyes are not sensitive to glycosylation patterns. Recently, imaging in combination with new techniques has been continuously applied. Haga et al. used azide-tagged GFP-labeled proteins to image cell-surface glycoproteins by Förster resonance energy transfer fluorescence microscopy.^471^ However, limited by the GFP protein, this method is unable to image endogenous glycoproteins. Belardi’s group has built on this foundation and achieved imaging of glycosylated forms of endogenous proteins using azidoglycan labeling and two-photon fluorescence lifetime imaging microscopy.^472^

Since most protein molecules are similar in size to nanomaterials, nanomaterials can be used as a backbone for the assembly of glycosylated probes, and glycosylation-modified fluorescent probes have been created.^473^ Zhang’s team designed nano-based metal-organic framework fluorescent probes with Zr (IV) and boric acid as active centers.^474^ Specific glycosylation sites were identified and in situ fluorescence imaging was performed by structural modification of metal-organic framework and alizarin red modulated fluorescence. Together, fluorescent labeling is hopefully to be widely promoted in glycosylation pattern identification due to its unique advantages of direct visualization and simplified operation procedures.

### Nanotechnology and other detection technologies

In addition to the common assays, there is a proliferation of novel techniques for the detection of glycosylation (Table 3). Maglia’s research group devised the nanopore method, which takes advantage of high ionic strength, and low pH environments and uses nanopores containing phenylalanine for identifying hydrophilic peptides and distinguishing between mono- and di-glycosylated peptides. The method enables qualitative detection and quantitative quantification of protein glycosylation.^475^

**Table 3:** Novel detection methods for glycosylation

	Methods	Principle	Advantages	Limitations	Reference
Separation enrichment affinity technique	LAC	Isolation and enrichment of intact glycopeptides is achieved by selective recognition of monosaccharides or glycans with specific structures by lectins immobilized on agarose or magnetic beads.	Widely used for the enrichment of specific *N*- and *O*-glycoproteins and glycopeptides. Multilectin affinity chromatography allows for the simultaneous enrichment of more diverse protein glycosylation by mixing multiple lectins.	Lectin specificity needs to be determined and multiple binding produces inertia.	^519–523^
	Hydrazine chemical enrichment	Trapping of glycopeptides by formation of covalent hydrazone bonds between the aldehyde group generated by oxidation of the glycan chain and the amino group on the hydrazide resin. Removal of the N-glycosyl chain by the PNGase F enzyme or chemical methods.	Simplified protein glycosylation and effective localization of *N*-glycosylation sites at the peptide level.	Complicated experimental procedures, especially the loss of glycan chain information.	^524^
	HILIC	A broad-spectrum enrichment method for the separation and enrichment of intact glycopeptides by the difference in polarity between glycosylated and non-glycosylated peptides.	Preserves the structure of intact glycopeptides. Enriches all types of intact glycopeptides without bias. Suitable for analyzing compounds in complex systems. Does not require expensive ion-pairing reagents and can be easily coupled with MS.	Co-expression of glycopeptides and non-glycopeptides leads to lower enrichment specificity.	^525–527^
	MAC	Glycosylated peptides are negatively charged and hydrophilic, which are selectively enriched by electrostatic interactions with metals.	High stability and low non-specific binding. Wide range of pH values. Multiple metal ions can be tested on the same resin to determine which metal ion is best suited for purifying the target protein.	Cannot be used on proteins that do not have a metal affinity. Some amino acids are susceptible to metal-catalyzed oxidation reactions leading to protein damage.	^528^
	Boric acid chemical method	Enrichment of intact glycopeptides using boric acid to form reversible covalent bonds with monosaccharides.	Captures and releases intact glycopeptides without affecting the structure of the glycan chain.	Different affinities between boric acid and different glycan chains.	^529–531^
Methods for glycoprotein identification/glycosylation site determination	Enzymatic method for PNGase F	Catalyze the non-lysosomal hydrolysis of an *N*(4)-(acetyl-β-d-glucosaminyl) asparagine residue into N-acetyl-β-d-glucosaminyl-amine and a peptide containing an aspartate residue.	Simplifies analysis of *N*-glycosylation. Good reusability and thermal stability.	Incomplete deglycosylation may lead to biased results.	^532,533^
	NMR	Caused by the spin motion of the nucleus.	Binding affinity and epitope information can be obtained. Analyze the structure, conformation and dynamics of carbohydrates and their interactions with biomolecular receptors such as proteins. Regardless of molecular size and without isotopic labeling.	Severe overlap of signal peaks, difficult resolution and low sensitivity. Multidimensional NMR requires milligram samples.	^466,534,535^
	Raman imaging	Utilizes spectral and spatial information to generate images.	A powerful visualization method with high sensitivity and specificity, without staining, with spatial and spectral resolution. Ability to record vibrational spectra in multiple regions to map the spatial distribution of glycans in human tissues.	Unable to quantify glycosylation levels. Detection of low abundance molecules requires next generation vibrating labels with higher sensitivity	^467,536^
Other detection technologies	Glycosylation-related gene testing	Using molecular biology and informatics techniques, the gene sequence of a glycan residue or glycosyltransferase is analyzed to derive a differential expression profile.	Suitable for the study of the specific structure of the lack of glycan, simple operation.	Technical and instrumental requirements.	^537^

*LAC* lectin affinity chromatography, *HILIC* hydrophilic interaction liquid chromatography, *MS* mass spectrometry, *MAC* metal affinity chromatography, *NMR* nuclear magnetic resonance

In addition to the methods mentioned above, there are also several new platforms being established. Some researchers have developed a software tool called GlypID 2.0, which incorporates CID and HCD information to achieve accurate characterization of *N*-glycosylation sites.^476^ A simplified GlycoSense method utilizes carbohydrate sensing microspheres assembled into a multi-suspension array to achieve the goal of simultaneous detection of multiple orthogonal glycosylation profiles simultaneously using common instrumentation.^477^ Blöchl et al. integrated glycomics with transcriptomics from public databases to unearth distinct glycochemical profiles based on FAB system classification.^478^ An anti-Tn antibody microarray platform was recently established for the detection of Tn+ glycoproteins in detecting sub-nanogram levels, which are almost universally present in cancer patients.^479^

## Conclusions and future perspectives

Glycosylation is an extremely complex and the most abundant type of PTM, which not only greatly contributes to the expansion of protein diversity, but also has far-reaching effects on protein functions. Glycosylation in the form of multiple glycan residues brings considerable changes to the proteome, and the myriad forms of polysaccharides or oligosaccharides linked together are a source of stereostructural diversity in proteins. Glycosylation is closely related to protein solubility, stability, and activity. For example, in the ER, the composition of glycans in the chaperone proteins calnexin and calreticulin tracks the folding state of the nascent polypeptide. *N*-glycosylation is localized on the side of the conserved structural domains of hepcidin, which regulates calcineurin secretion and promotes hepcidin maturation. If *N*-glycosylation occurs elsewhere in hepcidin, it misfolds and accumulates with calnexin over time.^480^

Abnormal or disturbed glycosylation cause human diseases. Congenital disorders of glycosylation are genetic disorders marked by hypoglycosylation of proteins and lipids, were first described in 1980 and are characterized by mutations in the gene encoding phosphomannanase.^481,482^ In addition, altered protein glycosylation is considered a hallmark of cancer. Glycosyltransferase GALNT7 alters *O*-glycosylation in prostate cancer cells and facilitates tumor growth.^28^ Unraveling glycan-based mechanisms in cancer can help decipher the molecular mechanisms of cancer biology.

New approaches are needed for early diagnosis, risk prediction and treatment of cancer, and glycans can be used to develop new non-invasive biomarkers. Some of the most commonly used serum biomarkers in clinical practice for cancer diagnosis and monitoring prognosis are glycoproteins. These include PSA, a major biomarker widely used in prostate cancer patients, CA125 in ovarian cancer patients, CEA in colon cancer patients, and CA199 in pancreatic cancer patients.^368,483,484^ Unfortunately, these glycoproteins are not exclusively found in one cancer type or not exclusively in one stage of the tumor. Finding the most specific and sensitive assay for detecting cancer then becomes a topic for research. For example, serum PSA testing for prostate disease has been around for a long time, but has led to problems of overdiagnosis and treatment. Researchers have studied the glycosylation patterns of PSA and found that a significant increase in core fucosylation with sialylation is more representative of prostate cancer.^485^

Combined protein binding and glycosylation pattern detection may provide better diagnostic and prognostic performance, but how one can accurately detects alterations in glycosylation sites and expression levels in clinic settings has become a major challenge. Advancement of detection methods for glycosylation patterns has been constantly pursued, from the initial MS and electrophoretic analysis to visualization imaging such as fluorescence and NMR, to the current gene detection technology and sophisticated data analysis platform. Increasingly, the latest advances in glycomics, proteomics, genomics, transcriptomics, and metabolomics data gathering and analysis are being used to better, understand how glycosylation regulates protein biological functions, and how new therapies can be developed by targeting glycosylation. Whether they are small molecule inhibitors targeting enzymes in the glycosylation process, or glycosylation-modified mAbs and novel vaccines, these modalities show the great potential to target glycosylation for therapy development. In conclusion, glycosylation serves as molecular “switches” to provide proteins or lipids with more diverse functions and play crucial roles in various aspects of life. Targeted therapies at glycosylation, either alone or in combination with more conventional therapeutics, have the potential to provide cancer patients and patients with diseases with better treatment options.
